# ‘Cyclical Bias’ in Microbiome Research Revealed by A Portable Germ-Free Housing System Using Nested Isolation

**DOI:** 10.1038/s41598-018-20742-1

**Published:** 2018-02-28

**Authors:** Alexander Rodriguez-Palacios, Natalia Aladyshkina, Jessica C. Ezeji, Hailey L. Erkkila, Mathew Conger, John Ward, Joshua Webster, Fabio Cominelli

**Affiliations:** 1 Digestive Health Research Institute, Case Western Reserve University School of Medicine, Cleveland, OH 44106 USA; 2 Division of Gastroenterology and Liver Diseases, Case Western Reserve University School of Medicine, Cleveland, OH 44106 USA

## Abstract

Germ-Free (GF) research has required highly technical pressurized HEPA-ventilation anchored systems for decades. Herein, we validated a GF system that can be easily implemented and portable using Nested Isolation (NesTiso). GF-standards can be achieved housing mice in non-HEPA-static cages, which only need to be nested ‘one-cage-inside-another’ resembling ‘Russian dolls’. After 2 years of monitoring ~100,000 GF-mouse-days, NesTiso showed mice can be maintained GF for life (>1.3 years), with low animal daily-contamination-probability risk (1 every 867 days), allowing the expansion of GF research with unprecedented freedom and mobility. At the cage level, with 23,360 GF cage-days, the probability of having a cage contamination in NesTiso cages opened in biosafety hoods was statistically identical to that of opening cages inside (the ‘gold standard’) multi-cage pressurized GF isolators. When validating the benefits of using NesTiso in mouse microbiome research, our experiments unexpectedly revealed that the mouse fecal microbiota composition within the ‘bedding material’ of conventional SPF-cages suffers cyclical selection bias as moist/feces/diet/organic content (‘soiledness’) increases over time (*e.g*., favoring microbiome abundances of *Bacillales, Burkholderiales, Pseudomonadales;* and cultivable *Enterococcus faecalis* over *Lactobacillus murinus* and *Escherichia coli*), which in turn cyclically influences the gut microbiome dynamics of caged mice. Culture ‘co-streaking’ assays showed that cohoused mice exhibiting different fecal microbiota/hemolytic profiles in clean bedding (high-within-cage individual diversity) ‘cyclically and transiently appear identical’ (less diverse) as bedding soiledness increases, and recurs. Strategies are proposed to minimize this novel functional form of cyclical bedding-dependent microbiome selection bias.

## Introduction

The importance of germ-free (GF) animals as a laboratory resource has exponentially grown with our expanded understanding of the complex role of microbes in disease modulation^1–7^ especially in the complex context of personalized diets, microbiome variability and genetics^8,9^. Although the use of GF mice in scientific publications has tripled over the last decade, GF facilities remain relatively scarce due to their high technical costs. Improving current GF research efficiency and experimental capabilities will allow more laboratories to adopt GF infrastructure to conduct more complex and parallel studies of diverse microbiotas^10^, as numerous diseases could be better treated with an improved causal understanding of microbes-diet-genomic interactions^11–13^. Novel complementary strategies to promote paralleled microbiome research are also needed since cross-contamination of cages in standard multi-cage pressurized isolators is a common and difficult problem to control when cages are enclosed together^14–16^.

Although mechanically pressurized ventilation with high-efficiency particulate arresting (HEPA) filtration have existed for decades in GF multi-cage isolation systems, and more recently in individually ventilated cages^17,18^, pressurized systems require anchored (nonmobile/nontransportable) infrastructure. Although all HEPA-pressurized isolators are ‘transportable’, arguably they cannot be moved freely by one person through elevators or stairs due to their large footprint and combined weight with their anchored ventilation systems. When occupied with mice, transportation of such isolators cannot be risk-free either without maintaining pressurized ventilation, because the lack of positive ventilation creates gaps in sterility barriers (*e.g*., back-flow of room air via negative pressurization induced by motion). To expand the current GF experimental efficiency of available systems^19,20^, we propose to use static ventilation for housing GF mice in individual GF cages that require no mechanical ventilation, pressurization, or HEPA filtration.

Herein we report the development of a validated, portable, static two-layer filtration static system that uses natural ventilation, where air flow is driven by within-cage differences in moist and air buoyancy^21^, for use during GF and parallel-microbiota transplant experiments in mice. Referred to as Nested Isolation (NesTiso, /*ne-stee-zoh/*), our design can be easily implemented and conceptually tested by assembling two static rodent cages of different sizes ‘one-inside-the-other’, with mice in the inner cage, resembling ‘Russian Nesting dolls’. Our NesTiso cages are portable since one person can easily transport and maintain them indefinitely without the need of positive ventilation, allowing the establishment and movement of GF animals or colonies outside of GF facilities for unprecedented experimental purposes.

The main objective of this report is to describe the housing design and test the hypothesis that NesTiso ensures long-term GF housing of GF mice. After two years of experimentation and handling of >45,000 mouse-days exclusively in NesTiso, we determined that the method is 99.9% isolation efficient, with as low as 0.10% risk of environment-to-cage contamination, and 100% capacity to prevent cage-to-cage dissemination of microbes. At the cage level, with a total of 23,360 GF cage-days (equivalent to maintaining one GF cage for 64 years), we determined that the cumulative probability of having a cage contamination event for each cage-opening (every 10 days) of NesTiso sets inside biosafety hoods can be identical to the probability of cage contamination inside multi-cage pressurized GF isolators (‘gold standard’ in this study). As an additional objective, we examined the effect of using NesTiso in mouse microbiome research using conventional mouse SPF feces and corncob bedding which is the most commonly used substrate for bedding material in laboratory rodent facilities. Microbiome experiments showed *(i)* that soiled (*i.e*., mixed with mouse excrements) corncob bedding material remarkably favors the enrichment of fecal murine *Bacillales*, *Burkholderiales* and *Pseudomonadales*, and *(ii)* that two different levels of bedding ‘soiledness’ can result in different fecal colonization patterns in GF mice, which combined represent a novel source of data variability and bias not currently accounted for in mouse research. Strategies are here proposed to minimize this novel functional form of Cyclical Bedding-dependent (CyBeD) Microbiome Bias in animal microbiome studies.

## Results

### NesTiso cage set design and thermography

**Figure 1:**
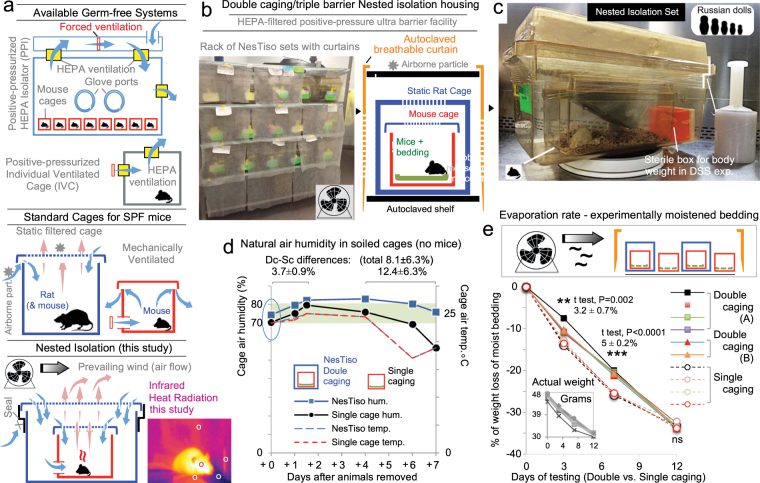
Nested Isolation System Design. (**a**) Illustration of ventilation and air filtration in housing systems commercially available for mice, and our Nested isolation system (NesTiso; non-HEPA air filtration occurs in inward/outward directions as air currents move by natural ventilation and external aeration). Mouse photograph, thermography demonstrates mice are a source of heat that instantly affects the temperature of the bedding material and surrounding elements via infrared rays reflectivity. Circles illustrate hottest spot near the eye (35.9 °C), and instant infrared reflection (heat radiation) that warms up surrounding surfaces (*e.g*., +2.9 °C on bench top; details in Supplementary Figs 1–3). (**b**) NesTiso setting in ultrabarrier GF room. (**c**) Germ-free NesTiso cage set in biosafety cabinet housing one 40 week-old GF-mouse during a 7-day DSS experiment (day 72 in NesTiso). Filter lids are sealed to cage bottoms using plastic wrap. Notice the space between the cages to store materials for individualized repeated aseptic handling and weighting of mice (small orange box). (**d**) Comparison of naturally occurring air humidity inside heavily soiled empty GF mouse cages monitored over time in NesTiso or standard single caging (NesTiso labeled as DC for double caging in illustration, and SC for single caging; 3 cages/group). Notice that NesTiso ventilation dynamics parallel that of SC. Air humidity differences were stable for four days and noticeable immediately after soiled cages were set as NesTiso (y-axis, oval). (**e**) Effect of external aeration with a household fan on the humidity (wet weight) of experimentally moistened soiled corncob bedding material (replicate sets A and B; without mice). Inset, actual bedding weight in grams (four replicas/cage) over time. Notice markedly improved ventilation and evaporation (bedding desiccation) in both NesTiso and SC. Paired-t test, 4–6-cages/4-replicas/cage.

To prevent contact with airborne particles (required for environmental exposure to microbes)^22–24^, NesTiso is technically a ‘double-caging/triple-barrier’ or ‘nesting 3-layer isolation’ system. Implemented using commercially available static cages, we housed cohorts of GF mice born in HEPA-pressurized isolators by placing the mice (**SAMP**1/YitFc [SAMP]^25,26^, C57BL/6 [**B6**], and Swiss Webster [**SW**]) inside mouse cages, and then nesting such cages inside larger rat cages. For air filtration, both nested cages had spunbonded polyester non-HEPA filter lids^22^, which were hermetically attached to the cage bottoms using stretch plastic film. As a third layer, NesTiso sets were placed on an autoclavable steel rack-cart safeguarded with breathable autoclavable curtains (Fig. 1a–c). Although mechanical ventilation efficiently exchanges air in standard cages, its use arguably causes cold-stress and immune-alterations in mice^27–29^. Natural ventilation in NesTiso is based on heat convection from the mouse causing infrared thermo-physical effects on the surrounding air. Thermal studies in mice^29–31^, architectural ventilation laws^21^, and NesTiso thermography (Supplementary Figs 1 and 2)^32^ indicate that cage air, if set at temperatures lower than that of the mouse, warms up by convection near the mouse via respiration or infrared reflectivity and rises, creating a (‘chimney effect’) column of air moving upward. Rising warm humid air currents then promote replacement with heavier colder clean air moving inward, causing passive air filtration as currents move in both directions through non-HEPA filters (Supplementary Fig. 3).

### External aeration improves natural ventilation

Because moist condensation was notorious in high animal density NesTiso cages (4–5 mice/cage), we quantified the air humidity in NesTiso, and the effect of external aeration on 7-day moistened-soiled bedding in empty NesTiso cages. Under laboratory conditions of stable air humidity (26.5 ± 4.89%) and temperature (23.8 ± 0.57 °C), natural air humidity in NesTiso was 3.7 ± 0.9% higher compared to static single caging (70.3 ± 1.5%), and cage humidity fluctuated in parallel over time in both NesTiso and single cages, indicating proper moisture exchange in NesTiso based solely on natural moisture-driven ventilation (Fig. 1d). Because external ventilation around the cages could improve air exchange with the cages’ interior, we then quantified if aeration of the cage-holding rack by using a household fan could improve natural ventilation, lowering the humidity within the NesTiso cages. Experiments on humidity of moistened corncob bedding demonstrated that external aeration was effective at reducing bedding moisture in the innermost cage. Measurement of moist bedding weight changes over a 12-day period demonstrated that aeration caused optimal steep evaporation curves of moist bedding in NesTiso (Fig. 1e), indicating that external aeration improves natural ventilation and bedding dehydration.

### Microbial screening confirms NesTiso GF status

Feed indwelling microbes that survive sterilization (gamma-irradiation, autoclaving)^14^, airborne particulates^22–24,33^, and human skin microbes are common sources of contamination of GF mice. To validate NesTiso as a GF system, we used fecal gram staining and quantified the test agreement between aerobic and anaerobic cultures to identify the most efficient microbial screening. For this purpose, we transported 32 GF mice in 19 NesTiso sets to a microbiology (non-GF, non-HEPA facility) laboratory, where cages were opened twice under a biosafety HEPA hood to feed the mice an SPF-grade irradiated diet over a 10-day period. Culture results showed that aerobic cultivation of feces correctly predicted the results (94.8%) of either mouse colonization with facultative anaerobes (only one cage had strict anaerobes), or the absence of microbes in GF mice by day 10 (Supplementary Fig. 4a–d). Kappa statistics^34^ confirmed optimal test-agreement (89.5%) between aerobic and anaerobic screening (Kappa = 0.78 ± 0.23; Z = 3.42; Prob > Z = 0.0003). The global performance of both cultures was further tested using receiver operating characteristic (ROC) regression and bootstrapping, where the probability of culture results from randomly selected contaminated cages (sensitivity) was compared to that of random non-contaminated cages (specificity)^35^, using as comparator both cultures interpreted ‘in series’^34^. ROC predictions showed that aerobic and anaerobic incubation have the same probability of differentiating GF from colonized mice (*P* = 1.0). Because most environmental contaminants are robustly aerobic, we recommend routine aerobic fecal cultures, incubation of soiled cages for fungi, fecal gram staining and weekly anaerobic cultures (Supplementary Fig. 4e,f). Mice were confirmed GF, on average, with nine negative tests. Serological health screening was also conducted in NesTiso mice confirming the absence of reactive antibodies and inadvertent exposure to 23 cultivable and uncultivable pathogens, including viruses^36–45^ (Methods and Supplementary Table 1).

### Containment of microbes and ‘quadrant infection control’ in NesTiso

Collectively, this study represents two years of monitoring mice for a total of >99,530 mouse-days, divided across three rooms (A, B, C). To determine if contamination events could be halted in NesTiso, we tested two strategies: In rooms A/B (~65 cages), in the event of a contamination we simultaneously tested and replaced all cages in the entire mouse colony, and eliminated all contaminated cages using an ‘all-in-all-out’ strategy; in Room C (~35 cages), without testing the entire colony, we only eliminated newly contaminated cages. Since implementation^14^, rooms A/B housed 62,780 mouse-days, of which, 40,880 were in NesTiso (twice the isolators’ capacity; ~23,360 cage-days, ~1,987 cage-openings; Table 1 and Supplementary Table 2). In average, NesTiso in rooms A/B (2.5 mice per cage; 26.9-week old) required over 1,381 routine fecal cultures and 300 cage-fungal incubations to monitor GF sterility. Only two cages were contaminated in room A (50 mouse-days), once with a fungus (*Penicillium* spp.), and 8 months later with a bacterium (*Bacillus* spp.). With ~1,987 cage-openings (in average once every 10 days), the risk of cage contamination with every opening was 0–0.1% (room A: 2/1,220; room B: 0/767). At the animal level, estimates indicate the daily risk of mouse contamination in NesTiso is 1 out of every 817 (50:40,830) days of housing using the ‘all-in-all-out’ strategy, which is longer than our oldest GF mouse born and housed in NesTiso (1.39 years of age). At the cage level, comparatively, NesTiso effectiveness was similar to managing cages in isolators, which had no contaminations in 2 years (0/548 cage-openings; 1-sided Fisher’s *P* = 0.61); however, NesTiso contaminations were restricted to affected cages (100% prevention), in contrast with reports of extensive dissemination of microbes across cages in isolators^14,15^. With 23,360 GF cage-days (equivalent to maintaining a GF cage for 64 years), the cumulative probability of having a cage contamination event for every cage-opening (every 10 days) of NesTiso sets inside biosafety hoods was statistically identical to that of opening cages inside the multi-cage pressurized GF isolators (2 events/1,971 openings vs. 0/548, two-tailed Fisher’s exact *P* = 1.0, Table 1).

**Table 1:** Two-year estimated contamination incidence of GF mice in Nested Isolation based on cage replacements every 10 days.

Mouse colony inventory *(22 month inventory snapshot)*	Cage counts^a^ Animal density			Age^b^ (weeks)		Estimated contamination incidence^cd^ (mouse-, cage-days, cage-openings)				
	cage count	adult (pups)	mice/cage	Adult mice mean ± SD	Oldest GF mouse	Contamt. cages Cumulative, n=	Cage-days	Mouse-days	Mouse-days per cage	Cage openings
B-Room 1–Isolator 1	4	11(5)	4.0	30.6 ± 5.8	45	—	2,920	11,680	2,920	292
B-Room 1–Isolator 2	4	10(5)	3.8	25.2 ± 6.0	34	—	2,920	10,950	2,738	292
B-Room 1–Isolator 3	3	8	2.7	23.9 ± 3.7	33	—	2,190	5,840	1,947	219
B-Room 2–Isolator 4	4	11(10)	5.3	25.5 ± 3.7	49	—	2,920	15,330	3,833	292
***Total Pressurized Isolator***	***15***	***40***	***2.7***	***26.3***	***49***	**0** ^**f**^	***10,950***	***43,800***	***2,920***	***1,095*** ^f^
B-Room 1–Nested Isolation	28	65(11)	2.7	24.9 ± 16.2	52	2	20,440	55,480	1,981	2,440
E-Room 2–Nested Isolation	21	47	2.2	29.9 ± 13.6	45	—	15,330	34,310	1,633	1,533
***Total NesTiso sets***	***49***	***112***	***2.3***	***27.4***	***52***	***2*** ^g^	***35,770***	***81,760***	***1,807***	***3,973*** ^g^
***Linear colony growth cumulative adjusted estimates for 2-y study AUC*** ^e^	***64***	***—***	***2.7***	***—***	***—***	***2***	***23,360***	***62,780***	***981***	***2,534***

^a,b^Isolators housed single static cages with young, active, or retired breeders ≤3) and pups. NesTiso cages were mostly used for nonbreeding mice. Totals (averages) for animal density and ages are based on adult mice data (no pups) to illustrate comparability of breeders with nonbreeding mice. Note that age in Nested isolation and Isolators are comparable.

^c^Mouse-days or cage-days = n of mice or cages × 730 days; mouse-days/cage = mouse-days ÷ n of cages; cage openings = cage-days ÷ days interval between cage replacement. The two contaminated cages occurred on two separate months of the study.

^d^Inventory snapshots of mouse colony in this experiment at months 8 and 22 were used for crude estimations for a 2-year period, assuming a constant number of mice and cages (see Supplementary Table 2 for comparison and timing of contamination events for the two snapshot inventories). Crude estimations of more realistic estimates were derived assuming a *linear growth of the colony reflecting an increment of the cage count of 2 cages per month, for a colony* expansion from 1 to 49 cages for months 0 to 22.

^e^A geometric estimating approach based on area under the curve is as follows: 64 cages × 2.74 mice/cage/day × 730 days × 0.5 AUC = 64,006 mouse days, which is similar to the 62,780 reported in the table. These approximations are conservative underestimating the actual efficiency of NesTiso in preventing new contaminations, and cage-cage contaminations, since we have at times housed larger number of cages in the study rooms.

^f,g^Statistically, the probability of having a contamination event in GF mice as a function of cage-openings across cages in isolators when compared to NesTiso sets was identical for the two methods (two-tailed Fisher’s exact statistics, *P* = 1.0).

Because simultaneous ‘all-in-all-out’ testing and cage replacement of an entire NesTiso colony can be stressful and laborious, we confirmed in room C (~35 cages, ~420 days, ~36,750 mouse-days) that eliminating only contaminated cages was ineffective at maintaining a low incidence of cage contaminations. We then validated that a NesTiso colony could be divided into quadrants for ‘all-in-all-out’ infection control (one quadrant/day; overnight disinfection), showing effectiveness comparable to the ‘all-in-all-out’ approach, while reducing technical stress.

### Long-term phenotypes, survival and breeding are unaffected by NesTiso

**Figure 2:**
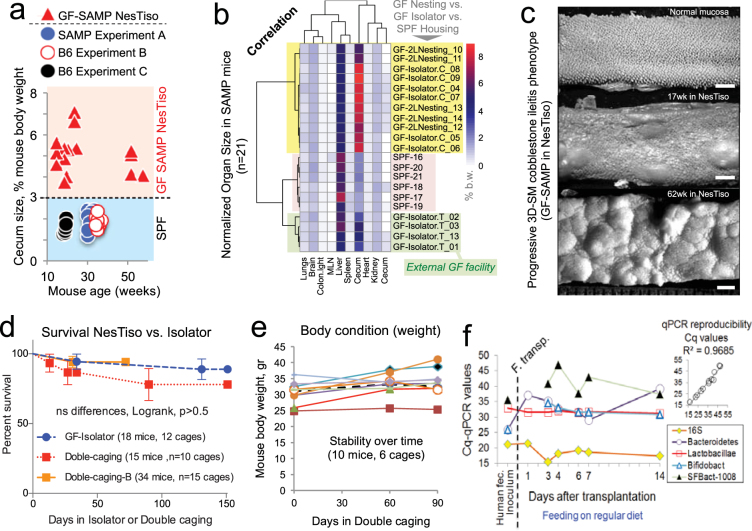
Nested Isolation has no negative impact on murine phenotypes. (**a**) The cecum size in NesTiso GF-SAMP mice is significantly larger compared to SPF mice as expected, and remains unaffected across ages (curve slope~0.01, *P* > 0.1; 3 experiments, triangles vs. circles, t-test *P* < 0.0001, n = 40). (**b**) Multivariable unsupervised cluster analysis of SAMP cecum and 8 other organs (normalized biomass, % of body weight) shows NesTiso mice (‘2LNesting’) are identical to mice raised in isolators in the same facility (Isolator.C). SPF and GF-isolator-T mice were included as external comparators. Mice from isolator T (Taconic, Inc.) clustered separately due to lower cecum size after transportation. Correlation statistics predict NesTiso and ‘Isolator.C’ cluster (*P* < 0.001, see univariate/hematocrit data in Supplementary Fig. 5a,b). (**c**) 3-D-stereomicroscopic profiling of the small intestinal mucosal surface illustrates the presence and progression of typical ileitis with ‘cobblestone’ lesions in NesTiso GF-SAMP. Scale bar, 1 mm. See histological features of cobblestone ileitis in Supplementary Fig. 5c–e). (**d**) Right-censored survival analysis (outcome variable: time to death) shows there are no differences on mortality incidence comparing GF mice raised in isolators vs. NesTiso. 72 and 151 day censored data (n = 67 mice, >40-wks old). (**e**) NesTiso has no negative effect on body weight as surrogate for animal welfare. Twelve-week-old SAMP mice gained or maintained weight as expected while housed for additional 12 weeks in NesTiso (1–2 mice/cage). (**f**) Quantitative 16s rRNA PCR enumeration of microbial abundance on the feces of a normal human donor and four transplanted NesTiso GF SAMP mice shows relative stability of bacteria families over time (1 mouse sampled per NesTiso set). Inset panel illustrates high qPCR test reproducibility. Abbreviations: Human fec., human feces; F. transplant, day of fecal transplantation; Bifidobact., bifidobacteria; SFBact-1008, segmented filamentous bacteria with 16S rRNA primer R-1008.

As alternative to measuring time-point cortisol levels as a measurement of animal adaptability, which induces stress to animals and increases the risk of microbial contamination in GF mice during handling, we monitored murine morphological and breeding phenotypes to determine whether NesTiso is suitable for the study of long-term phenotypes. One of the functions of gut commensals is to aid in digestion and modulate tissue morphology. By comparing organ dimensions from mice housed in GF-NesTiso, GF-isolators and SPF conditions, we determined that the organ biomass and hematocrit (as surrogate for dehydration and erythrocythemia) of GF-NesTiso mice were similar to that of GF mice in isolators in comparison to SPF mice (Fig. 2a–d). We then assessed whether NesTiso caging affected the spontaneous intestinal disease phenotype in SAMP mice, and found no effects on the natural three-dimensional occurrence of Crohn’s disease–like ileitis (cobblestones)^26^ lesions in GF-SAMP mice, GF-SAMP survival (five-month), or the SAMP body weight (three-month) after transplantation with normal human fecal microbiota (Fig. 2c–e and Supplementary Fig. 5)^46^. Furthermore, NesTiso did not induce signs of systemic, integument, or intestinal diseases in ileitis-free GF-B6 and GF-SW mice. A four-week breeding trial, conducted by cohousing males and females (2:3/cage; 2 cages/strain) for three days, also determined that breeding yields using NesTiso for the three mouse strains were ranked as expected after 30 days. SAMP mice were the least productive strain (one pup from 1/6 females); B6 were intermediate (8 pups from 1/6 females), and SW were the most productive (67 pups from 6/6 females; Fisher’s *P* < 0.05). Long-term survival in NesTiso was further documented in this study by breeding and maintaining GF mice for as long as 72 weeks of age, when animals were removed solely for experimental purposes or died due to aging-associated complications. Other studies have followed GF animals outside isolators only for 2–3 or 12 weeks^19,20^.

### Human fecal microbiota transplants to mice in NesTiso

The containment of microbes in NesTiso makes the system ideal for studying the stability and colonizability of human fecal microbiota transplants (FMT) in GF mice. Because FMT-mice often require BSL-2 isolation in facilities housing SPF-mice, we tested the portability of NesTiso FMT-mice to a BSL2-room, sharing biosafety hoods with 20–30 SPF-cages. We determined whether 12-week-old mice would have stable FMT microbiota in NesTiso, and whether NesTiso FMT-mice would have 16S rRNA gene microbiome signatures of SPF-mice. Fecal DNA and quantitative real-time PCR analysis of four 16S rRNA-universal and -specific bacterial taxa primers (*Lactobacillaceae*, *Bacteroidaceae*, *Bifidobacteriaceae*, segmented filamentous bacteria)^26,47^ showed that FMT in GF-SAMP was stable over 14 days in NesTiso (Fig. 2f).

**Figure 3:**
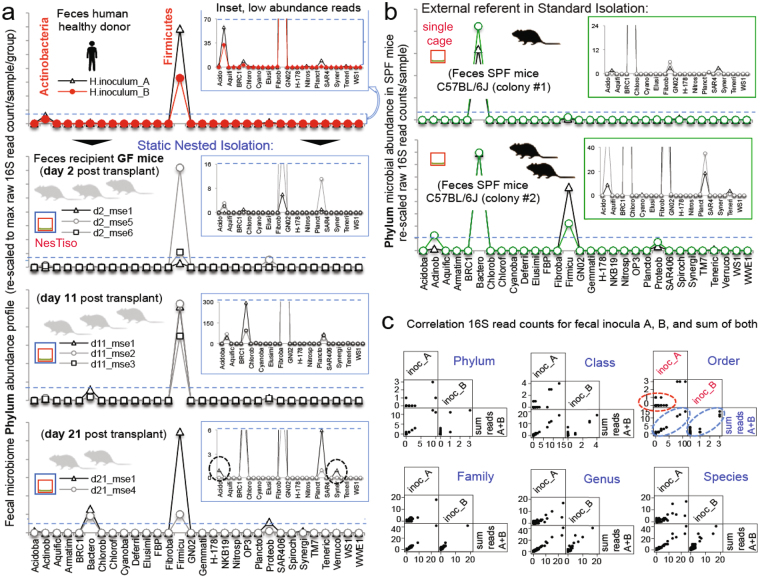
Fecal microbiome signatures of donor, transplanted and other mice support NesTiso microbe containment in BSL-2 facility. (**a**) Microbiome ‘*phylum signatures*’ profile for replicated samples from the feces of a normal human donor, and below, the corresponding fecal profiles of GF-SAMP mice that received the donor sample as transplant (FMT) on day 1 (10 mice transplanted; 4 NesTiso sets, to diminish within-mouse repeated measures data dependency 8 collected frozen fecal samples were randomly selected for analysis, since individually ventilated cage data has earlier shown appropriate clustering over time^15^). Normalized 16S rRNA gene (microbiome) abundance of fecal bacteria at the phylum level (Y-axis), for all 31 possible phyla in this experiment (X-axis). Notice similar quantitative *Firmicutes*-rich signatures of donor and FMT mice over time (binary data, 6/6 of 31 possible taxa present), suggesting FMT colonizability and stability in NesTiso. The slight increase in *Bacteroidetes* on day 21 compared to day 2 and 11 suggests natural enrichment in mice possibly due to different diet and digestive biology compared to humans. (**b**) Fecal microbiome *phylum signatures* of SPF B6 mice (used as external comparator) are richer in *Bacteroidetes*. Binary probability statistics (presence/absence) for all phyla observed in FMT (including low abundant phyla circled in inset plot Fig. 3a on day 21) and SPF mice, indicates that each group had unique signatures and that cage-cage cross-contamination in FMT studies is unlikely using NesTiso (considering the limitations of microbiome data, this data is supported by the 100% prevention of cage-cage dissemination of microbes in NesTiso; see text). (**c**) Correlation of raw 16S rRNA gene read counts in the feces of the human donor in panel 3a, for two technical aliquots (A and B) to that of the sum of A + B reads (interpreted ‘in series’). Notice that low abundant read data distribution improves the linearity when reads in A and B are added (see diagonal ovals in ‘Order’ panel; Supplementary Fig. 6).

Slightly extending the study period to 21 days to encompass the establishment of adaptive immunity, 16S rRNA microbiome analysis of fecal samples that were randomly collected from 10 mice sampled on days 2, 11 and 21 after FMT showed that FMT-mice in NesTiso had the healthy profile of the human donor (6/6 of 31 possible phylum taxa) that was rich in *Firmicutes*, while conventional concurrent SPF-mouse signatures in the same facility were distinct and rich in *Bacteroidetes* (Fig. 3a,b). These 16S rRNA microbiome data further support NesTiso as a suitable portable caging system that can be used to prevent cage cross-contamination^14,15^, facilitating the parallel study of diverse microbiotas and their effects on transplanted GF mice. By categorizing read count data as binary (presence/absence), and using probability-of-recovery statistics (% of mice transplanted having the taxa), we also noticed that two analytical replicas interpreted ‘in series’ (sum of taxa reads in both replica) normalize the distribution of low abundant taxa making it preferable over interpretation ‘in parallel’ (only taxa positive in both replica), or using single aliquots. When using NesTiso in FMT experiments, it is thus also advisable to submit ≥2 donor aliquots for microbiome sequencing and interpret these profiles ‘in series’^34^ (Fig. 3c and Supplementary Fig. 6). Also, results suggest that is not advisable to exclude taxa with ‘lower number reads in a sample’, as occasionally recommended in bioinformatic pipelines, but rather to consider using NesTiso to prevent contamination and analyze all available high-quality data ‘in series’.

### NesTiso-independent enrichment of fecal *Bacillales, Burkholderiales* and *Pseudomonadales* in soiled bedding

**Figure 4:**
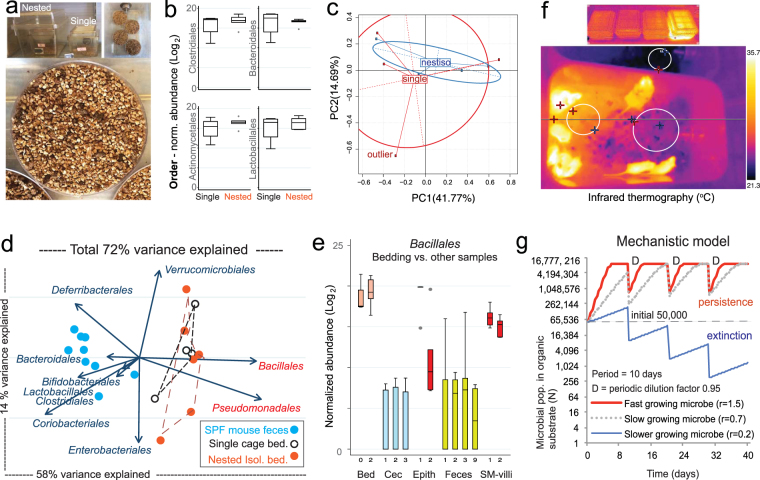
NesTiso does not bias the fecal microbiome, but enrichment of fecal *Bacillales* and *Pseudomonadales* in mouse bedding reveals mechanistics of novel form of ‘cyclical microbial bias’ in microbiome research. (**a**) Split-plot experimental design to assess effect of NesTiso on fecal microbiome. Moist corncob bedding with SPF SAMP-mouse feces was randomly divided into petri dishes, and incubated inside either ‘Single’ or ‘NesTiso’ static cages at 23 °C for 28 days (4 dishes/cage; 4 single vs. 6 NesTiso cages). (**b**) Box plots with 16S rRNA microbiome read abundance of four bacterial Orders (*Clostridiales*, *Bacteroidales*, *Actinomycetales*, and *Lactobacillales*) from pooled bedding material shows no difference between Single and NesTiso cages after incubation (n = 4 vs. 6 cages, t-test *P* > 0.05). Notice consistently reduced data variability (standard deviations) in NesTiso. (**c**) Multivariate principal component analysis (PCA) of 16S rRNA microbiome bacterial orders illustrate NesTiso does not affect the microbiome of mouse feces in bedding material compared to single caging. (**d**) Comparative biplot microbiome analysis of mouse bedding and fecal samples illustrates *Bacillales* and *Pseudomonadales* [and *Burkholderiales* in Supplementary Fig. 7] as major orders enriched in bedding material independently of caging type (explaining 58% of data variance, x-axis). *Clostridiales* and other anaerobes in left side of biplot cannot grow aerobically. (**e**) Normalized box plot of mouse microbiome data from feces, cecum content, intestinal tissues and bedding samples to contextualize the comparable enrichment of *Bacillales* in both bedding and intestinal villous samples in mice. (**f**) Infrared analysis of cages housing conventional SPF-mice illustrates that the temperature in the cage bedding can be as high as 31 °C (mouse max = 37.4; outside cage min = 21.3), which could favor the enrichment of fast growers introducing cyclical selection bias favoring suitable aerobic microbes in mouse microbiome research. (**g**) Mathematical modeling and simulations over several dilution events using a mechanistic event-customizable model^49^ predicts that fecal microbial persistence or extinction in the mouse cage bedding depends on the speed of growth of each fecal microbe in the bedding over cumulative dilutional cage replacement events (set in the model as mouse bedding cage replacements every 10 days; see script and cyclical Periodicity Rules in Supplementary Materials).

Because NesTiso may increase air humidity if not aerated, we hypothesized that FMT studies performed in NesTiso could favor the selection of certain fecal microbes compared to conventional single caging. This was important as we noticed some contaminants thriving in soiled-humid bedding, while others (slow-growing environmantal fungi) unexpectedly disappeared from GF mice in dry (frequently-replaced) cages. In split-plot experimentation, we determined that the DNA microbiome profile of a freshly soiled SPF-SAMP bedding mixture (split into 40 petri dishes) was identical for NesTiso and single caging after incubation for 28 days at 23 °C, indicating NesTiso double caging did not contribute to microbial bias (Fig. 4a–c). Collectively, however, bedding microbiomes were significantly enriched with *Bacillales, Pseudomonadales* and *Burkholderiales* when compared to fecal mouse microbiome studies from conventional single cages (Fig. 4d and Supplementary Fig. 7). More relevant, an expanded comparison showed that coincidentally the same orders (*Bacillales* and *Pseudomonadales)* were markedly enriched in stereomicroscopically dissected mucosal-associated microbiomes^48^, rising concerns for the first time about potential bias driven by the cyclical selection and enrichment of fecal microbes in soiled bedding (Fig. 4e and Supplementary Fig. 7). Interestingly, we have identified in our facility and during this study *Bacillus* spp., *Staphylococcus petrassii/aureus*, *Paenibacillus woosongensis*, and *Pseudomonas alcaligenes* as GF contaminants, supporting the relevance of both *Bacillales* and *Pseudomonadales* enrichment, survival and adaptability to the bedding material and housing conditions in laboratory animal research.

### Modeling and predictions of bacterial growth and extinction over cyclical enrichment-dilution events

The enrichment of certain microbes in the bedding material might depend on the type of substrate and lead to cyclical changes in the cage microbiome as cages become warm, humid and rich in organic matter over time (Fig. 4f). Quantitation revealed that the organic ‘nutritious’ enrichment in the bedding has linear dependence on animal density, ‘bedding cycle’ interval (clean bedding becomes soiled, then replaced with new bedding usually 7–10 days), and grinding behavior (*e.g*., by day 10, a 5-mice-bedding contains 6.9% feces and 43.6% diet). To visualize the periodic dilutional effect of cage replacements at fixed intervals on microbial selection (both survival and extinction), we implemented a mechanistic mathematical model using a logistic function validated for bacterial growth in liquid medium coupled with a customizable event function accounting for periodic dilution events (as surrogate for bacterial and organic substrate replacement), using ‘deSolve’^49^ to ran simulations in open-source R software. Simulations illustrated how fast-growing microbes, depending on their rate of growth, persist in the model over several cycles, while slow-growing microbes become extinct (Fig. 4g), and importantly allowed the recognition of unaccounted periodicity mechanisms influencing cyclical microbial selection herein refer to as basic Cyclical Bedding-dependent Microbiome Periodicity Rules (see brief description in Supplementary Materials). With the mathematical visualization of differential microbial selection over time and recurrent dilution events (cage replacements)^49^ and inferred predictions, we next tested experimentally whether bedding soiledness influenced the gut microbiota profile directly in mice.

### Co-streaking culture assay reveals bedding soiledness cyclically influence the gut microbial profile

**Figure 5:**
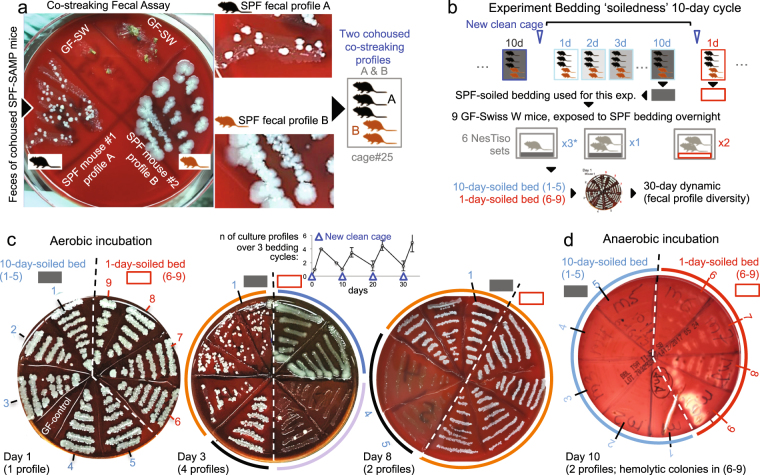
Exposure of GF mice to different SPF bedding ‘soiledness’ results in distinct colonization patterns and cyclical bedding-dependent (CyBed) microbiome variability in the mouse gut. (**a**) Semi-quantitative fecal culture (‘co-streaking’) assay illustrating two distinct fecal culture profiles of five littermate SAMP mice cohoused for 20 weeks. TSA blood agar, aerobic, 37 °C, 5d. See appearance after 36 h of incubation, and follow up gram stain of fecal smear in Supplementary Figs 8 and 9, respectively. Fecal enumeration and single-colony Sanger sequencing indicates abundant cultivable microbes contribute major fractions of bacterial DNA in mouse fecal microbiome. Under the assumption that cultivable and uncultivable microbes interact dynamically, the assay serves to monitor the comparative dynamics of fecal systems. (**b**) Experimental design to determine the effect of soiledness on colonizability differences in GF-SW mice, and the dynamic effect over three cage replacements. (**c**) Aerobic incubation of ‘co-streaked’ fecal samples illustrates cultivable microbiota differences. Notice ‘co-streaking’ fecal profiles of 9 SW mice (labels, 1–9): mice look similar on day 1; then appear more distinct with 4 cultivable profiles on day 3; then similar on day 8 (two profiles). Inset line plot, number of ‘co-streaking’ fecal profiles over 33 days (3 bedding cycles). Notice pattern of ‘co-streaking’ fecal profile variability oscillates cyclically over time with every new cage change (more alike when beddings are 10-day-soiled; more distinct when samples are collected three days in clean cages, *i.e*., 3-day-soiled). (**d**) Anaerobic hemolytic (virulence) fecal profiles on day 10. Notice that 4 mice exposed to 1-day-soiled bedding have abundant hemolytic anaerobes (absent in 10-day-soiled bedding mice). Exposure to variably soiled bedding affect collective virulence profile of acquired/transmissible microbes from bedding. Because microbiota abundance and virulence variation may influence animal phenotypes, it is necessary to control for CyBeD microbiome variability to improve scientific rigor during experiments, but also during breeding since newborn pups from a single colony may be variably imprinted by the cyclically biased bedding microbiome.

We hypothesized that GF-NesTiso mice exposed to 1-day-soiled SPF-bedding would have a different transmissible fecal microbiota profile compared to mice exposed to 10-day-soiled SPF-bedding, and that over time their individual profiles would periodically vary with every bedding cycle. Because microbiome assays and data analysis are not real time and can be time consuming and technically intensive, we developed a rapid culture assay of feces (streaked on TSA blood agar, incubated overnight; herein referred to as ‘co-streaking’, the assay results are reproducible across fecal pellets) that facilitated the enumeration of colony types and thus the cost-effective assessment of gut microbial dynamics in near real time. By streaking the feces of ‘co-experimental’ mice on the same agar plate, our ‘co-streaking assay’ became a semi-quantitative screening tool to visualize the periodic dynamics of the gut microbiota (Fig. 5a and Supplementary Fig. 8). Remarkably, we found in a 30-day (3-bedding-cycles) experiment that overnight exposure of GF-SW (healthy) mice to the bedding of SPF-SAMP (ileitis-prone) mice yielded persistently different co-streaking patterns depending primarily on whether the mice were exposed to either 1-day-soiled beddin﻿g or 10-day-soiled bedding (Fig. 5b–d). As hypothesized, co-streaking also showed more diversity (colony types) across cages and mice on day 3 after cage replacement, which remarkably disappeared (less diversity, primarily same colony type) by days 8–10, a phenomenon that recurred with every bedding cycle. We also noticed that within cages, some animals cyclically exhibit their own pattern of microbiota profile (individuality), which were markedly influenced (cyclically disappeared) as cages became soiled (Fig. 5b–d). Confirming the high occurrence of within-cage individualities, a cross-sectional screening of 80 adult SPF (AKR, B6, B6^TNFdeltaARE/+^, SAMP) mice in 45 cages (without controlling for bedding soiledness) revealed that up to 70–82% of cages cohousing >2 genetically-identical mice had >1–2 individual co-streaking patterns, which contradicts the perception that cohoused SPF mice have the same microbiome profile (Fig. 5 and Supplementary Figs 8 and 9), identifying an new unrecognized form of microbiome intra-cage variability despite presumed homogeneous coprophagic behavior^50–54^ and rising questions about cohousing as a preferred design in mouse microbiome research^52,55–57^, especially since cohousing also alters numerous phenotypes of interest (*e.g*., metabolism, obesity, inflammation)^58–61^. A parallel shotgun metagenomic profiling study of fecal samples from male-female breeding pairs cohoused for 30 weeks since weaning (3 mouse lines) confirmed that although cohoused mice clustered together (predicting cage allocation) in an ‘unsupervised Euclidean heat map analysis’, binary (yes/no) discordance analysis showed that cohoused mice vastly differed in the number of detectable fecal bacterial families that the paired mice did not share in each cage (up to 33% discordant, not attributable to sex; mean 24 ± 5%; 51 bacterial families in the study)^9^. As an alternative approach, we have developed a protocol where all experimental mice are not cohoused (because studies require large number of animals and since an ideal cohousing design would be impossible^56^, not simple^62,63^ and IACUC approved maximum animal density is 5 mice/cage) but instead *(i)* gavaged a composite of their collective fecal microbiota^26^, *(ii)* allowed to establish a baseline collective microbiome, and then *(iii)* followed up to determine the functional relevance of microbiota that animals select as experiments progress^8,9,26,64,65^. In the context of well-known long-term microbiome stability and the stable core microbiome in humans^66^, our findings also rise questions about whether the assumption of ‘difficult-to-control’ temporal microbiome stochastic variability reported in mice^15,67^ is truly biologically correct, or whether such ‘temporal variability’ represents the distribution of study results randomly confounded by an unrecognized technical artifact that occurs when the timing of animal sampling for microbiome analysis in not controlled and accounted for as a function of bedding microbial selection. Therefore, we further assessed experimentally the effect of various degrees of bedding soiledness on the competitive growth and 9-day survival of three cultivable abundant fecal microbes, representing distinct bacterial families detectable in SPF mice.

### Dose-effect study illustrates soiledness favors gut *Enterococcus faecalis* over *Escherichia coli* and *Lactobacillus murinus*. 

**Figure 6:**
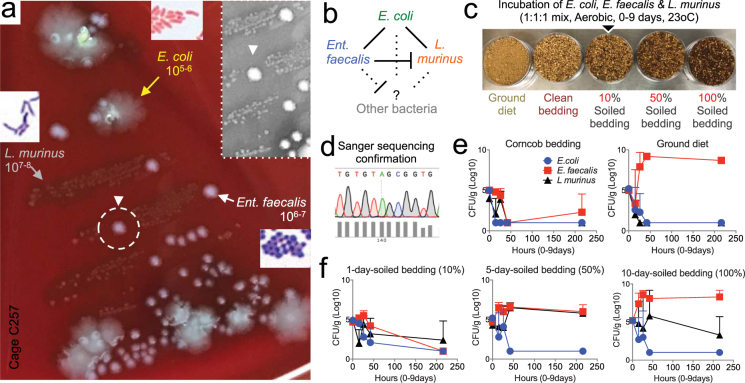
*In vitro* growth of three abundant fecal aerobic bacteria in bedding shows *Enterococcus faecalis* resilience to soiledness. (**a**) Close up photograph of a TSA agar streaked with feces of AKR mouse after 48 h of aerobic incubation 24 h, 37 °C. Notice three abundant distinct colonies (spreading, *Escherichia coli*; white, *Enterococcus faecalis*; grey, *Lactobacillus murinu*s) that could be semi-quantitatively ranked and compared. Notice *E. faecalis* inhibition over *L. murinus* when in close proximity (dashed circle/inset close-up). Gram-stain morphologies are shown. (**b**) Schematic of observed interactions among the selected microbes in TSA and other hypothetical uncultivable microbes. (**c**) Design of *in vitro* experiment to determine if a mixture of three bacteria could equally grow on bedding at different concentrations of GF-soiled bedding, clean bedding and diet. Inoculated bedding samples were incubated at 23 °C for 9 days. (**d**) Sanger sequencing chromatograph of bacterial DNA samples from selected and enumerated isolates confirms enumeration data. (**e**,**f**) Line plots illustrate that when incubated as a 1:1:1 mixture, *E. faecalis* is highly resilient to soiledness, and able to readily grow on the GF-grade rodent diet used. Unexpectedly, *E. coli* was the least adaptable fecal microorganism in the cage environment. Biologically and experimentally relevant, *L. murinus*, a worldwide aerobic species is best adapted to 5-day-soiled bedding, indicating selection bias favors its abundant growth until it is inhibited by the overgrowth of *E. faecalis* towards bed-day 10 (Supplementary Fig. 10). These findings derived from SPF-AKR mice confirm the cyclical predictions illustrated in panels 6b-c, which derived from interpretation of the fecal co-streaking profiles of the SPF-SAMP microbiota that was transmisible to GF-SW mice.

Various bedding substrates are available for use with rodents, including corncob, paper products, aspen wood chips, cotton and grass fiber pellets; however, animal welfare regulations recommend bedding that allows foraging, burrowing, digging, nest building and absorbs urine, ammonia, humidity and feces^68–70^. Because autoclaved corncob is an efficient common bedding material used for the routine rearing of laboratory rodents^68^, we next tested and confirmed *in vitro* that the amount of ‘soiledness’ influences the microbial selection of highly abundant (10^5^–10^8^ CFU/g) gut aerobes inoculated in autoclaved corncob bedding. Experimentally, three distinct fecal bacterial ‘co-streaked’ types from a healthy SPF-AKR mouse (shiny-spreading *Escherichia coli*, small-gray *Lactobacillus murinus*, domed-white *Enterococcus faecalis* which inhibits *L. murinus* when in proximity) were added as a 1:1:1 mixture to NesTiso-petri dishes containing either sterile clean bedding, GF-10-day-soiled bedding from a NesTiso cage, a mixture of clean bedding containing 10% or 50% of the GF-soiled bedding (as surrogates for 1- and 5- day-soiled bedding based on mathematical model), or GF-diet. Remarkably, bacterial enumeration on TSA over time (23 °C for 9 days) demonstrated that each bedding condition result in very different bacterial growth ratio profiles (different from 1:1:1 inoculated ratio), favoring in most cases the enrichment/selection of *E. faecalis* in soiled cages. Intriguingly, plain GF-diet as growing substrate inhibited and disfavored the survival and growth of otherwise fast-growing *E. coli* and *L. murinus*, suggesting that certain types of (digested or indigested) diets might further favor bedding-enriched *Enterococcus faecalis*, arguably in the most proximal segments of the mouse gut (Fig. 6). With the abnormal abundance of *L. murinus* in experimental environments, it is reasonable to expect that such aerobic microbe could influence the mouse physiology, as it has been demonstrated that its overgrowth causes biotin-dependent alopecia in mice^71^. On the other hand, the overgrowth *E. faecalis* can selectively inhibit a large number of other (mainly gram-positive) microbes via bacteriocin-like inhibitors greatly common amongst the family *Enterococcaceae*, within the order *Lactobacillales*.

## Discussion

To date, with over 100,000 mouse-days of data and experimentation, this report illustrates the effectiveness of NesTiso as a portable GF animal housing alternative to pressurized systems. Major advantages of NesTiso include its potential for cost-effective scalability, and the elimination of risks associated with back-flow ventilation problems and sterility-barrier failure, widely documented across isolation units in positive-pressurized ventilation hospital settings^72^ (also very likely to occur in GF facilities). Since implementation, NesTiso has allowed the portability of GF animals across research facilities, and improved efficiency in parallel gnotobiotic/FMT studies. As NesTiso implementation could ‘democratize’ GF-grade capabilities, our results provide immediate support to ongoing government efforts to improve rigor and reproducibility in microbiome research, as well as efforts by the World Health Organization^73^ to promote natural ventilation for hospital infection control. Further, due to major human gut microbiota variability, studies with broad coverage of donors and large number of GF animals and cages are needed to elucidate the mechansisms of establishment of human-derived microbiotas in mice, and their effect on biology and disease phenotypes. In this context, NesTiso has the potential to further improve research efficiency by preventing the risk of environmental transfer of microbes between transplanted mice, which is known to confound research by affecting mouse metabolic traits when inadvertent contamination occurs^16^.

After the implementation of NesTiso, we have not identified major disadvantages to justify working solely with multi-cage isolators again. In some GF facilities, the replacement of soiled cages inside isolators is conducted by replacing only the dirty bedding material from each cage, and not the entire cage set. To improve efficiency, sterile material is entered into the isolators together with food, and other supplies in bulk to expedite husbandry. Since the risk of cross-contamination of cages and the whole isolator is high when only the bedding material is replaced^14^, in our facility we replace the entire cage set by moving the mice to a new clean cage every time we change cages, in both, isolators and NesTiso. In this comparable context, NesTiso has some technical advantages compared to isolators: *(i)* the speed of husbandry flow is faster in NesTiso with more cages changed per unit of time since it is easier to work under biosafety hoods than inside isolators, *(ii)* the number of animals housed per floor area is higher since animals can be housed in cages stacked vertically and require less space for transfer chambers or for operators using the glove ports needed in front of each isolator, *(iii)* there are no maintenance costs associated with technical inspection and service of positive pressurized equipment, *(iv)* dirty soiled or microbially contaminated or purposely gnotobiotic cages are easily handled and disposed very efficiently in NesTiso since there is no need to move cages in or out of transfer compartments, *(v)* the initial investment to begin a GF colony with NesTiso is virtually null because there is little to no need to purchase or invest on specialized equipment, and *(vi)* there is no noise or vibration disturbance associated with ventilation equipment with passive ventilation in NesTiso. Currently, NesTiso settings are being improved to further minimize area footprint and to maximize natural ventilation. Following strict surgical grade aseptic protocols widely available in the literature and successfully applied for surgery in GF mice^74^ we anticipate that other laboratories could implement and expand successfully their GF research portfolio using NesTiso.

This report also highlights that important cyclical alterations exist within the fecal microbiome profile of experimental mice, presumably due to the selective enrichment of specific aerobic microbes within the mouse bedding material. If ignored, cyclical bedding-dependent microbiome alterations could have unpredictable confounding effects on the interpretation of phenotypic results across numerous fields of murine research, including the unpredictable consequences of microbiome imprinting of newborn pups in colony-breeding programs. As a potential solution, we earlier developed a fecal homogenization protocol^26,64^, where all experimental animals housed in various cages are exposed to the entire pool of gut microbes harbored in the feces of all experimental mice and the bedding of all cages. Herein, we propose to apply that protocol only with fresh feces (and not bedding material), and to further improve scientific rigor by either considering designing novel cage flooring systems to prevent the permanent contact of mice with their feces and soiled bedding (which does not occur in humans); or by conducting experiments with frequent cage replacements and proper ventilation accounting for animal density to minimize humidity/soiledness, and collect samples for investigational purposes 2 days after animals have been transferred to new bedding/cages. Although we did not test all potential combinations and possibilities (*e.g*., animal density, body weight, drinking, grinding behaviour, etc), results indicate that microbiome experiments would benefit if conducted with cages having comparably reduced animal density (*e.g.,* 2 mice/cage), with animals being sampled for analysis on day 2 post-cage replacement (*e.g.*, ‘2 × 2 cage sampling rule’). Unless this is accomplished, each study should examine and control for the effect of cyclical bedding microbiome selection (which may vary widely) on target investigational phenotypes. Together, our data also indicate that mouse cohousing^52,55–61^ might not be necessarily robust in all scientific scenarios to control for microbiome variability in mouse research, unless we control for bedding-dependent cyclical microbiome selection bias, and intra-cage sustained mouse-mouse gut microbiota variability. Strategies have long been explored to prevent coprophagia in nutritional studies since the 1960s^50,75,76^. Simpler practices here described, however, could control for bedding soiledness as a potential source of cyclical microbial bias in modern mouse microbiome research.

## Materials and Methods

### Animals and germ-free facility

The portable static isolation strategy herein proposed was tested by housing inbred GF SAMP1/YitFc (SAMP) and C57BL/6J (B6) mice and outbred Swiss Webster (SW) mice re-derived or obtained from Taconic Biosciences Inc. (Hudson, NY). All mice were maintained as GF colonies at the Animal Resource Center (ARC) at Case Western Reserve University School of Medicine (CWRU). SAMP mice are a sub-strain of AKR/J mice originally developed in Japan that spontaneously develop intestinal and extra-intestinal inflammatory disease^25,26,77,78^, and has a polygenic genotype^79^. GF positive-pressurized HEPA rigid isolators (Plas-Labs Inc™ HEPA filtered isolation glove boxes with maximum capacity of 12–14 cages; 4–5 mouse cages/isolator, 1–5 adult mice/cage) were located inside an ultra-barrier pressurized HEPA-grade facility. Each GF isolator allowed for the manipulation of mice and supplies via four sets of permanent gloves and a port of entry, which was opened as needed, usually once a week. Animals were housed in wire-topped polycarbonate shoebox cages (~30 cm L; 15 cm W; 15 cm H) in a 12 h:12 h light:dark cycle. Autoclaved GF-grade 40–50 kGy irradiated pellet food (PMI Nutrition Int’l., LLC., Labdiet® Charles River. Vac-Pac Rodent 6/5 irradiated, 5% kcal% fat) or autoclaved (Prolab RMH 3000; porcine animal-derived fat preserved with BHA; 6.8% content by acid hydrolysis) diets and water in bottles were provided *ad libitum*^26^. Portability experiments where NesTiso cages were taken out of the ultrabarrier facility were conducted in BSL-2 grade laboratories equipped with standard HEPA filtration vent systems on the ceiling, but were not positively-ventilated or pressurized representing most standard clean laboratories. In those settings, HEPA-filtered air was readily available in biosafety cabinets which were used to open and replace the cages. Protocols on animal handling, housing, and transplant of human microbiota into GF mice were approved by the IACUC and the Institutional Review Board at CWRU, in accordance with the National Research Council Guide for the Care and Use of Laboratory Animals^70^.

### Nesting cages: static double-layer isolation setting and thermography

Cages and materials used are commercially available to assure results are generalizable to other laboratories. In brief, referred to as ‘double-caging/triple-barrier’ or ‘nesting 2-layer isolation’ (NesTiso), the proposed housing strategy was tested by housing cohorts of GF mice (produced in standard GF isolators) inside autoclaved static mouse cages^80^, which were then placed (nested) inside larger rat static cages (Allentown Inc., Allentown, NJ; see Results and Fig. 1 for details). Animals and cages were microbiologically monitored and handled by trained personnel under strict GF-grade aseptic conditions and our routine GF practiced following stringent disinfection protocols using complete isolation-grade fabric impermeable gowns, double gloves, hairnets and masks, or N95 respirators when deemed medically appropriate for personnel desiring not to be exposed to disinfectant vapors^14^. Thermography infrared image analysis in mice and cages was conducted using standardized principles^29–32^ and a thermal camera (FLIR E95 with Intelligent Autofocal^TM^ Optics) with capability to measure 161,472 point (range, −20 to +1500 °C) temperature pixels allowing allows sensitive detection of spatially confined minute thermal differences (464 × 348 native resolution, spectral range 7.4–14 µm). Differential quantitation of selected areas was conducted using the proprietary thermography camera software (FLIR tools for Mac^TM^ v.2017).

### Animal handling and disinfection

Disinfection protocols to ensure aseptic environmental conditions were based on quaternary ammonium-based soap to remove organic matter, 70% ethanol to remove grease and dehydrate; and Spor-Klenz^®^ (Steris Corp., Groveport, OH, 6525; 1% hydrogen peroxide, 10% acetic acid, 0.08% paracetic acid) on rust-sensitive equipment^14^. Floors and other surfaces were disinfected with Spor-Klenz^®^ and Clidox^®^ (Pharmacal Research Laboratories, Inc., Waterbury, CT, 96120F, chlorine dioxide). Biosafety hoods equipped with new HEPA filters and sterilized daily or weekly with chlorine gas or Spor-klenz vapors were used whenever cages or animals were manipulated (*e.g*., feces collection, body weight measurements). Autoclaved sterile gowns and hairnets, masks (N95 or cartridge half-face piece) and impermeable plastic sleeves were worn by personnel to prevent exposure of the NesTiso cage sets and animals to human dust or microorganisms, and to reduce personnel exposure to disinfectants.

### Husbandry and sanitation

Although the deleterious effects associated with ammonia are critical in conventional mice, ammonia is not relevant in GF animals (due to lack of urea-utilizer, ammonia-producing gut microbes). For sanitation purposes, replacement of whole NesTiso cage sets under GF or fecal microbiota transplant experiments followed comparable regulatory guidelines for conventional housing^81^, which is daily monitored by the CWRU ARC personnel and IACUC committee which monitors husbandry compliance with the NRCG-CULA. NesTiso sets were replaced every 7–14 days based on animal density, production of soiled material, and animal grinding behavior^82,83^. Every cage was routinely replaced under biosafety cabinets at least once weekly for animal densities of 3–5 mice/cage, and once biweekly for 1–2 mice/cage. In compliance with static cage usage for conventional (SPF-microbiota) mice, we used corncob bedding due to its absorbent capacity to lower air humidity inside cages^68,84^. This bedding material has been shown to minimally influence mouse body core temperatures compared to other materials^29^. In all cases, animals were handled using Spor-klenz disinfected, or autoclaved and rubberized 12-inch long forceps.

### Microbiological monitoring of GF status and cage-cage cross contamination

All mice inside both pressurized isolators and NesTiso sets were routinely tested using standard culture-based microbiological procedures and gram-staining^85^. Culture of feces and cage bedding material was conducted aerobically and anaerobically (10% CO_2_, 10% hydrogen, 80% nitrogen) using Tryptic Soy Agar (TSA) supplemented with 5% of defibrinated sheep blood. Luria Bertani, de Mann Rogose Sharpe, and McConkey agars were also used (Becton, Dickinson and Company, Franklin Lakes, NJ). Nutritious brain heart infusion broth supplemented with 5% yeast extract was used to test feed sterility and rule out bacterial contamination as needed. To monitor the risk of fungal contamination, we tested selected cages at 1–3 week intervals using fresh feces and direct plating onto potato dextrose agar (PDA), sabouraud, and Candida chromID agars (Oxoid, BBL, bioMérieux SA, France; 30 °C, 7 days). In addition, we also incubated 20–100% of soiled cages after adding 100 ml of water from the drinking water bottle (23 °C, aerobically, 21 days) to allow for fungal spore germination and the formation of vegetative aerial colonies, which aid in the confirmation and taxonomic classification of fungi^14^.

In a culture-independent manner, we also gram-stained mouse feces to verify that animals were not colonized *in vivo* by microorganisms that may be uncultivable using the *in vitro* methods described^85^. An expert board-certified microbiologist, who could distinguish microbes from dietary vegetable fibers, intestinal epithelial cells, inflammatory cells, and dye crystals and artifacts, conducted the interpretation of gram stains. If analysis revealed the presence of suspect microorganisms, animals were quarantined and gram stained and re-cultured 1–2 days later to verify mouse colonization (as indicated by an increased number of CFU and gram-stained microbes). Three consecutive negative gram stains or culture results were needed to declare a suspect NesTiso cage as free of germs (GF), based on infectious guidelines in veterinary medicine where horses with infectious agents (i.e., *Salmonella* spp.) require between three to five consecutive negative cultures to deem a horse free of the pathogen^86,87^. Our data indicate that two consecutive negative results are optimal to prove the mice were GF, and as such is an approach we use before enrolling any GF mouse cohort into experimentation. PCR was not used to test GF mice, although a qPCR-amplicon RFLP method has been recently validated for GF testing^85^, since DNA of dead and food indwelling microbes could not always be differentiated from active colonization and because PCR has been shown to be less sensitive than culture and gram-staining in identifying intestinal colonization in gnotobiotic mice and poultry^88,89^. Microbial DNA was also extracted from single purified colonies on TSA or PDA agars using the QiaAmpFast DNA extraction kit (Qiagen, City, ST) with some modifications (bead-beating with Sigma-Aldrich 500-µm beads, MP Fastprep-24 homogenizer; 1000 RMP 2 runs of 20s; AS lysis buffer). Microbial identification was based on single colony PCR amplification and Sanger sequencing, using 16S rRNA sequencing of V1–2 regions and Earth microbiome primers 515F/860R^90^. Ribosomal internal transcribed spacers 1 (ITS-1) and 2, and the 5.8S rRNA regions were sequenced for fungi using ITS1 (5′TCCGTAGGTGAACCTGCGG) and ITS4 (5′TCCTCCGCTTATTGATATGC) primers^14,91^. Species designation was based on NCBI Bacterial 16S rRNA and the fungal UNITE databases using BLASTn^92^.

### Cage air humidity and evaporation of soiled bedding experiments

We hypothesized that adding an extra layer of static filtration around the static mouse cage would presumably reduce ventilation exchange^80^, increasing humidity accumulation measured using digital monitors of air humidity and temperature (AcuRite 00613). Therefore, our first experiment involved the qualitative evaluation of water condensation within the cages with and without external ventilation (by using a 20 cm diameter table fan set two meters from the cages, ~1750 revolutions/minute). We tested three conditions (SPF, GF-isolator and GF-NesTiso) and measured (%) air humidity changes over a 7-day period of time inside mouse-free cages that had soiled bedding after housing five mice per cage for 7 days^80^. Lastly, we quantified the rate of evaporation of soiled moist bedding (weight changes) over a 12-day period (longer than the 7 days recommended for regular husbandry of static cages), with and without ventilation. Experiments on cage humidity were conducted without mice to minimize uncertainty due to animal behavior (urine production, grinding). Experiments were conducted in a laboratory with stable room air temperature (23.8 ± 0.57 °C) and relative air humidity (26.5 ± 4.89%).

### Mouse intestinal disease phenotype and survival analysis

To understand the effects of NesTiso on maintaining mouse phenotypes, we used SAMP mice, which display a well-characterized intestinal inflammation phenotype with 100% penetrance that resembles the typical three-dimensional (3D) cobblestone lesions of Crohn’s disease. Body weights was used as an indicator of animal health and welfare, and was monitored beginning in 10-week old mice (n = 10) for 90 days after their introduction to NesTiso. Post-mortem histological and stereomicroscopic 3D-pattern profiling^26^ were conducted on terminal ilea to assess the persistence of the Crohn’s-like intestinal phenotype in NesTiso cages. In another experiment, we compared mean cecum size (cecum weight ÷ body weight ratio* 100) among mouse cohorts, since GF mice have relatively large ceca due to absence of microbiota. For this purpose, adult (>14 weeks old) GF mice in NesTiso were compared to GF-SAMP mice in isolators, SPF-SAMP mice, and second mouse line prone to developing Crohn’s-like ileitis (B6^TNFare^)^26^. To determine if NesTiso increased the risk of mortality in SAMP mice, we compared the natural mortality across cohorts of GF mice housed in NesTiso or GF-isolators for up to 6 months using survival analysis.

### Fecal material transfer experiment

To determine the suitability of NesTiso for housing moderate densities of mice that harbor gut commensal microbiota (3–4 mice/cage, without external ventilation), we conducted a humanized fecal matter transplant experiment with 10 GF SAMP mice using frozen feces of a healthy (40-year old) human donor. All methods were carried out in accordance with guidelines approved by CWRU Institutional Review Board. Samples were obtained from the Cleveland Digestive Diseases Research Core Center Biorepository, which is also IRB approved, and which obtain the informed consents from all donors of fecal matter following strict regulations. We manipulated the mice weekly for fecal collection, and monitored the stability of the transplanted microbiota in fresh murine feces at 2, 11 and 21 days post-transplant by performing qPCR to determine the relative abundance of five bacterial families^26^. 16S microbiome analysis of fecal DNA samples from three mice for each time point was conducted by amplifying the V1-V3 regions using Illumina Truseq and HiSeq. 4000 protocols. Bioinformatics analysis was conducted using Greengenes and default Qiime pipelines (http://qiime.org).

### Soiled bedding microbiome analysis

To determine the effect of NesTiso on the 16S microbiome profiles, dry sterile dry corncob bedding material was experimentally inoculated with SPF mouse feces (20% of dry bedding weight), moistened with distilled water (25% volume/dry bedding-feces weight; ml/g), homogenized, and divided into aliquots that were placed in 10-cm sterile petri dish bottoms to achieve ~1 cm-thick layers (46.5 ± 2.28 grams of bedding/petri dish). Bedding humidity was adjusted to reach water content comparable to levels in naturally soiled bedding material of cages with breeding mice (*i.e*., 25% of bedding moisture relative to autoclaved dry corncob bedding in cages with three adult breeders and one-week old pups) after 7 days of housing in GF isolators. After 21 days of incubation of five dishes/cage, inside each of six NesTiso sets and four standard static mouse cages (23 °C, no external ventilation), bedding material was examined *in situ* for enumeration of fungal colonies and homogenized to extract a pooled sample of DNA for 16S microbiome analysis.

### Serology to assess inadvertent exposure to common rodent pathogens

Because certain pathogens (e.g., viruses and *Mycoplasma pulmonis*) cannot be detected by the described culture-based methods^33^, we also collected serum samples from six sentinel GF mice that were housed for six to twelve months in NesTiso to confirm the absence of exposure to 23 rodent pathogens. Fresh sera collected from euthanized mice were independently submitted by veterinary personnel at our Animal Resource Center-CWRU for testing at an external diagnostic institution (IDDEX Laboratory, Worthington, OH). Concurrent testing of other SPF rodent colonies from our institution served as test controls.

### Breeding potential of acutely humanized GF mouse lines in NesTiso

We next tested breeding and early nursing capabilities of mice housed in NesTiso by comparing the breeding efficiency of GF-SAMP with that of commercial GF-B6 and GF-SW 12-week-old mice transplanted with human gut microbiota. Based on our records, predicted breeding efficiency would rank SAMP mice as the poorest breeders, followed by B6 mice, and then SW mice with the highest number of viable healthy nursed pups by 1 week of age. Following oral gavage with a 400 µL aliquot of human gut microbiota, nine 10-week old mice were housed in NesTiso sets (5 mice/cage; 2 sets/strain; at 2:3 male:female ratio) and left to mate for 3 days; males were then removed from the cages. The number of pups produced per pregnant dam was determined 30 days after animals were set to breed.

### Effect of exposure of NesTiso GF mice to soiled bedding of SPF SAMP mice

To determine the potential impact of mouse exposure to different degrees of soiled bedding material^33^ on the gut microbiome, nine 20-week-old GF SW mice were exposed overnight to bedding from five SPF 19-week-old SAMP mice. SPF bedding originated from a single cage housing a cohort of five SPF-SAMP mice. The bedding material from the SPF cage was sampled at the nesting site and on wetter sites on days 1, 3 and 10 for culture and DNA microbiome analysis. The remaining bedding for days 1 and 10 were homogenized manually (separately) within the cage and aliquoted to be used as SPF-bedding for the cages that would house the GF mice. In average, each GF mouse was exposed to 40 grams for approximately 22 hours. Mice were assigned to either 1-day- or 10-day-SPF bedding a priori in sets of 1, 1, 1, and 2; and 2 and 2 for the 10-d and 1-day SPF bedding aliquots, respectively. After the exposure period, mice were transferred to GF-NesTiso sets, and feces were collected for culture and DNA extraction for microbiota culture assays. To prevent confounders, mice were not handled for the following three days (NesTiso cages were sealed and maintained at room temperature), when fecal samples and bedding material were collected for culture and DNA extraction, and mice initially caged singly were re-cohoused together as a trio. Thereafter, during the follow-up phase of the experiment, animals were monitored either in 2 pairs as initially set for 1 day-SPF bedding; and 1 pair and a trio for the 10 day-SPF bedding material. During the following 10-day cage changing cycles, the mice and bedding were sampled on days 3 and 8–10-day post cage change, for three cycles. Analysis of culture data derived from streaking fecal samples on TSA agar was conducted to assess the dynamics of the cultivable fecal microbiota over time. After incubation at aerobic and anaerobic incubation, photographs were taken, and representative colony phenotypes were selected for each fecal profile for sub cultured for purification and Sanger sequencing for species identification as above described.

### *In vitro* experiment for enumeration of a microbial cocktail in bedding material

By using NesTiso, the three most abundant bacteria in the co-streaking feces of cohoused SPF-AKR/J mice, and 10-fold serial dilutions in PBS with enumeration in TSA, we quantified to what extent bacteria would grow in NesTiso petri dishes containing moist soiled bedding material. Single colony PCR identified the most abundant aerobic bacteria in the AKR fecal sample as *Enterococcus faecalis*, *Lactobacillus murinus* and *Escherichia coli*. After purification and subculture, we determined that the bacteria in (1:1:1) cocktail experiments orally administered by esophageal gavage to three GF 20-week old SAMP mice (10^6,7^ CFU/mouse in 400 uL of phosphate buffered saline) reproduced the proportions of the 20-week-old donor AKR/J mice (10:1:1). In split-plot experimentation, then we simultaneously inoculated the same 1:1:1 mixture to 5 different sterile substrates (clean sterile corncob bedding, ground GF irradiated autoclaved diet, and three concentrations of soiled bedding; see experimental designs in Fig. 6b). The substrates were aerobically incubated in petri dishes within NesTiso sets for 9 days at 23 °C.

### Microbiome analysis

Fecal and bedding microbiome analysis was conducted with sufficient coverage to infer the presence or absence of abundant taxa and to quantify the risk of cross-contamination of transplanted mice with murine SPF microbiota at the phylum level (2–3 Log_3_ range difference between 100-bp pair-end reads of most and least abundant bacteria in sample). Total read counts for samples in were approximately 2500 and 25,000–40,000 reads per sample for Figs 3 and 4, respectively. Binary interpretation of phylum data (presence/absence) indicated that *(i)* recipient mice had a microbiome binary profile (‘phylum signature’) that was virtually identical to that of the human donor for at least 21 days indicating microbiome colonizability/stability. DNA extraction was conducted using Qiagen reagents (Tissue and blood kit). Library preparation and 16S rRNA microbiome sequencing and primary analysis was conducted using MiSeq Illumina protocols and bioinformatics standard pipelines based on Qiime at the Beijing Genomics Institute in Shenzen, China. Statistical analysis of OTU normalized 0.00017 + log2 transformed data tables was conducted using STATA v13.0 and R software v. 3.4.0 packages.

### Mathematical modeling

The mechanistic exploration of the microbiome driven hypothesis was conducted using available mathematical modeling functions for discontinuous logistic growth of populations with discrete events in R software (R-project, Vienna, Austria) package ‘deSolve’^49^. This package contains modules that allow the incorporation of customizable dilution simulation dynamic events to differential equations. The rationale and detailed description of a novel set of mathematical rules governing the periodic dynamics of cyclical microbial bias inferred from mechanistic interpretation of simulated data are described in detail and supported with additional references in Supplementary Materials (section Mathematical Visualization of Cyclical Bedding-Dependent Microbial Growth and Cumulative Selection, Microbiome Periodicity Rules and R-Script).

### Statistics

Body weight curves and normally distributed continuous parameters were tested using repeated measures (area under the curves, or univariate sum statistics of paired data points as recommended^93^) and parametric t-test statistics. When assumptions were not fulfilled, nonparametric methods were used^34^. Right-censored survival analysis data was conducted by computing survival fractions using Kaplan-Meier statistics^46^. Point wise 95% confidence intervals of survival fractions were computed using the log-log transform approach. An alpha level of 0.05 was considered in all cases significant. 95% confidence intervals are reported as primary measure of data dispersion to aid in the interpretation of the p values if larger than 0.05 and lower than 0.1. STATA (v.13; College Station, TX, USA), R (R-project, Vienna, Austria), and Graph Pad Prism (La Jolla, CA, USA) software were used for statistical analysis and graphics.

### Ethical IRB and IACUC approvals

This study was carried out in accordance with the recommendations and principles set by the National Centre for the Replacement, Refinement and Reduction of Animals in Research. Experiments and protocols were approved by the IACUC and the Institutional Review Board (IRB) at Case Western Reserve University. Fecal specimens from humans were obtained following protocols and personnel approved by the IRB who obtained informed consent from adult participants that donated specimens for testing in mice.

### Declaration of data availability

Data and detailed protocols are available upon request or freely available as Supplementary Materials.

## Electronic supplementary material

Supplementary Information
