# Supplementary material for: ‘Cyclical Bias’ in Microbiome Research Revealed by A Portable Germ-Free Housing System Using Nested Isolation
Source: Sci Rep. 2018 Feb 28;8:3801. doi: 10.1038/s41598-018-20742-1 (PMC5830500; doi:10.1038/s41598-018-20742-1)
Supplement: Supplementary file 1 — Supplementary Information [file 41598_2018_20742_MOESM1_ESM.pdf]

'Cyclical Bias' in Microbiome Research Revealed by A Portable Germ-Free Housing System Using Nested Isolation

Alexander Rodriguez-Palacios [1,2*], Natalia Aladyshkina [1,2], Jessica C. Ezeji [2], Hailey L. Erkkila [2], Mathew Conger [1,2], John Ward [1,2], Joshua Webster [1,2], and Fabio Cominelli [1,2]

[1]Digestive Health Research Institute, Case Western Reserve University School of Medicine, Cleveland, OH 44106, USA; [2]Division of Gastroenterology and Liver Diseases, Case Western Reserve University School of Medicine, Cleveland, OH 44106, USA

Supplementary Information

- **Supplementary Figures**
  - **Suppl. Fig. 1.** Mouse infrared heat radiation and reflectivity on different materials.
  - **Suppl. Fig. 2**. Movement of heat radiation, 'chimney effect', and potential applications.
  - **Suppl. Fig. 3**. Close-up photographs of the cage lid filters used in NesTiso.
  - **Suppl. Fig. 4.** Microbial Screening of GF mice and Portability of Nested Isolation Sets.
  - **Suppl. Fig. 5.** Organ dimensions, hematocrit and ileitis phenotypes are not affected by NesTiso.
  - **Suppl. Fig. 6**. Analysis of 16S rRNA microbiome recovery probabilities after microbiota transplantation is preferable to be conducted interpreting microbiome sequence reads in series without eliminating taxa/samples with low counts.
  - **Suppl. Fig. 7**. Microbiome of bedding samples in the context of other samples also illustrates enrichment of *Burkholderiales*.
  - **Suppl. Fig. 8.** Fecal samples illustrated on Figure 5a after 36h of incubation on Tryptic Soy Agar supplemented with 5% of defibrinated sheep blood.
  - **Suppl. Fig. 9.** Gram stain of fecal sample illustrates distinct cohoused gut microbiome profiles are long lasting.
  - **Suppl. Fig. 10**. Growth and survival of three abundant fecal murine bacteria incubated in various (corncob bedding:diet) substrates.
- **Supplementary Tables**
  - **Suppl. Table 1.** Pathogens Tested by Serology in Germ-Free Mice in Nested Isolation.
  - **Suppl. Table 2.** Two-year estimated contamination incidence of GF mice in Nested Isolation based on cage replacements every 10 days and two mouse colony inventory snapshots.
- **Supplementary References**
- **Supplementary Information on Mathematical Modeling**
  - Supplemental material and additional references on Mathematical Visualization of Cyclical Bedding-Dependent Microbial Growth and Cumulative Selection: Microbiome Periodicity Rules and R-Script.

---

*Corresponding author:
Alexander Rodriguez-Palacios
DVM, MSc, DVSc, PhD. Dipl. ACVIM, Dipl. ACVM
Assistant Professor of Medicine
Case Western Reserve University School of Medicine
Office: (216) 368-8545; Fax: (216) 368-0647
E-mail: axr503@case.edu

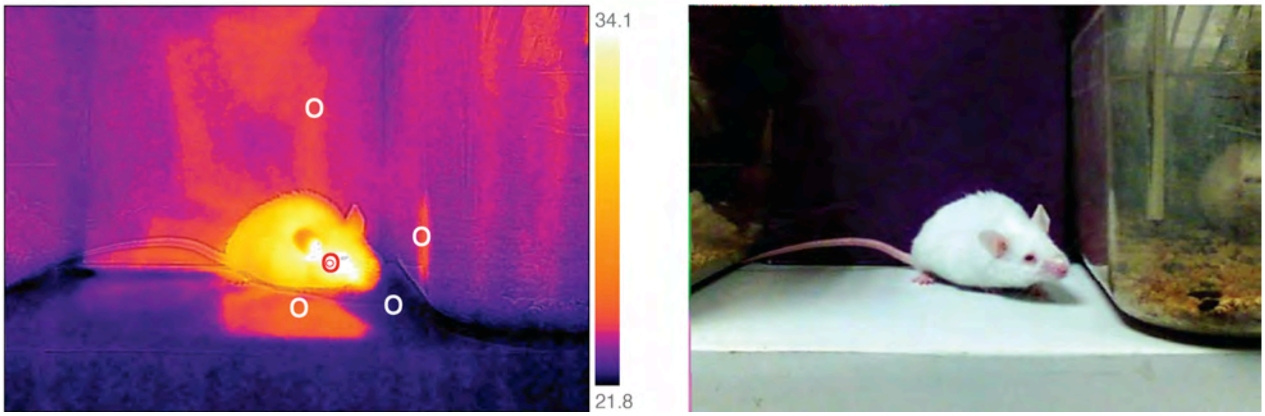

Supplementary Figure 1. Mouse infrared heat radiation and reflectivity on different materials.

Paired thermography and digital images (left and right panel, respectively) demonstrate mice are a source of heat that affects the temperature its surroundings via infrared rays reflectivity (heat radiation). Mouse is standing on a laboratory bench-top (nonreflective-to-light) surface between two rodent cages (right and left) and a paper box (in the background). Circles illustrate hottest spot near the eye (35.9°C), and infrared reflection (25.3°C) on the cold (22.4°C) bench-top and cage wall (24.3°C). Notice silhouette of heat reflection from researcher's body (located at ~1m) on background (nonreflective-to-light) paper box (24.3°C). Room air temperature, 23.0°C.

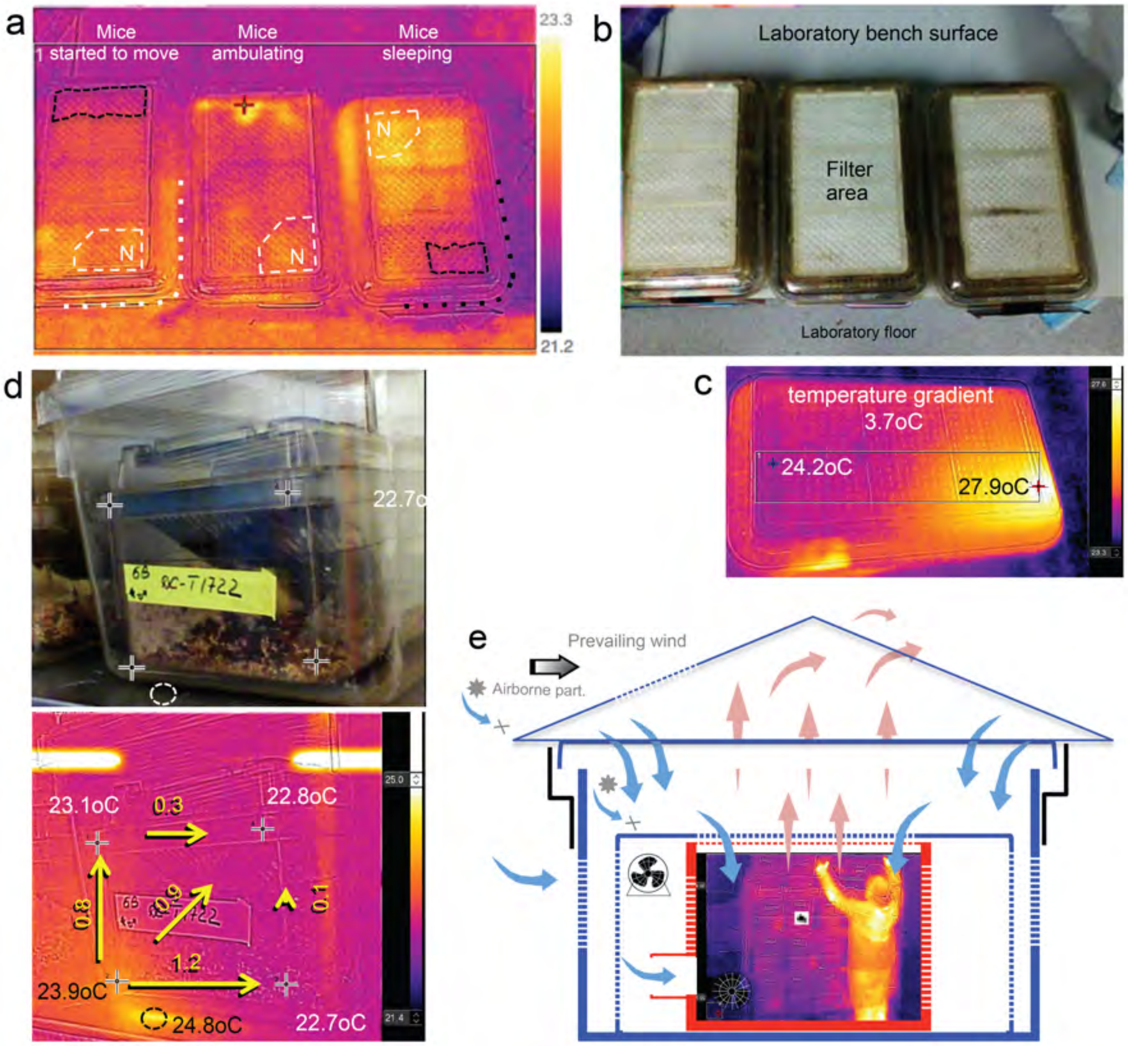

Supplementary Figure 2. Movement of heat radiation, 'chimney effect', and potential applications.

a-b) Aerial view of mouse cages (filtered lids seeing from above) occupied by mice who are housed at the center of nested isolation (NesTiso). Notice the hot spots (heat radiation) on the filter lids. Mice were in a dark room sleeping in their nests. The letter N at the center of dashed areas indicates the location of the nests. Images were taken early in the morning within 2 minutes of cage transportation which made the mice to awake and move around. Quantitative thermal analysis shows the colder areas (thick dotted black polygons/lines) are opposite to the nesting site illustrating the 'chimney effect' upward movement of warm air from the mice (heat source). The cage at the center has a mouse that moved out of its nest and stopped to groom on the opposite side of the cage where the hot spot is indicated by a '+' sign (hottest spot in the cage). **c**) Thermal irradiation differential on the lid between the coldest and hottest areas is as little as 3.7^{o}C with two 12-week-old male GF mice sleeping on their nest (located on the right lower corner of the cage; '+', hottest spot in cage). **d**) Thermal irradiation differential on the sidewall of the outer NesTiso cage set. Notice that the mouse-housing cage at the center of NesTiso irradiates heat that warms up the sidewalls of the second outer cage wall, which illustrates temperature gradients that promote ventilation between the two nested cages in NesTiso. Notice heat from the nest also increased the temperature of the metallic rack shelf, that held the NesTiso set overnight, in proximity to the mouse nest (the dashed circle on the metallic shelf is the image's hottest spot; '+' signs are for spatial reference. **e**) Nested isolation/triple barrier principles could be applied to other fields of research/building engineering.

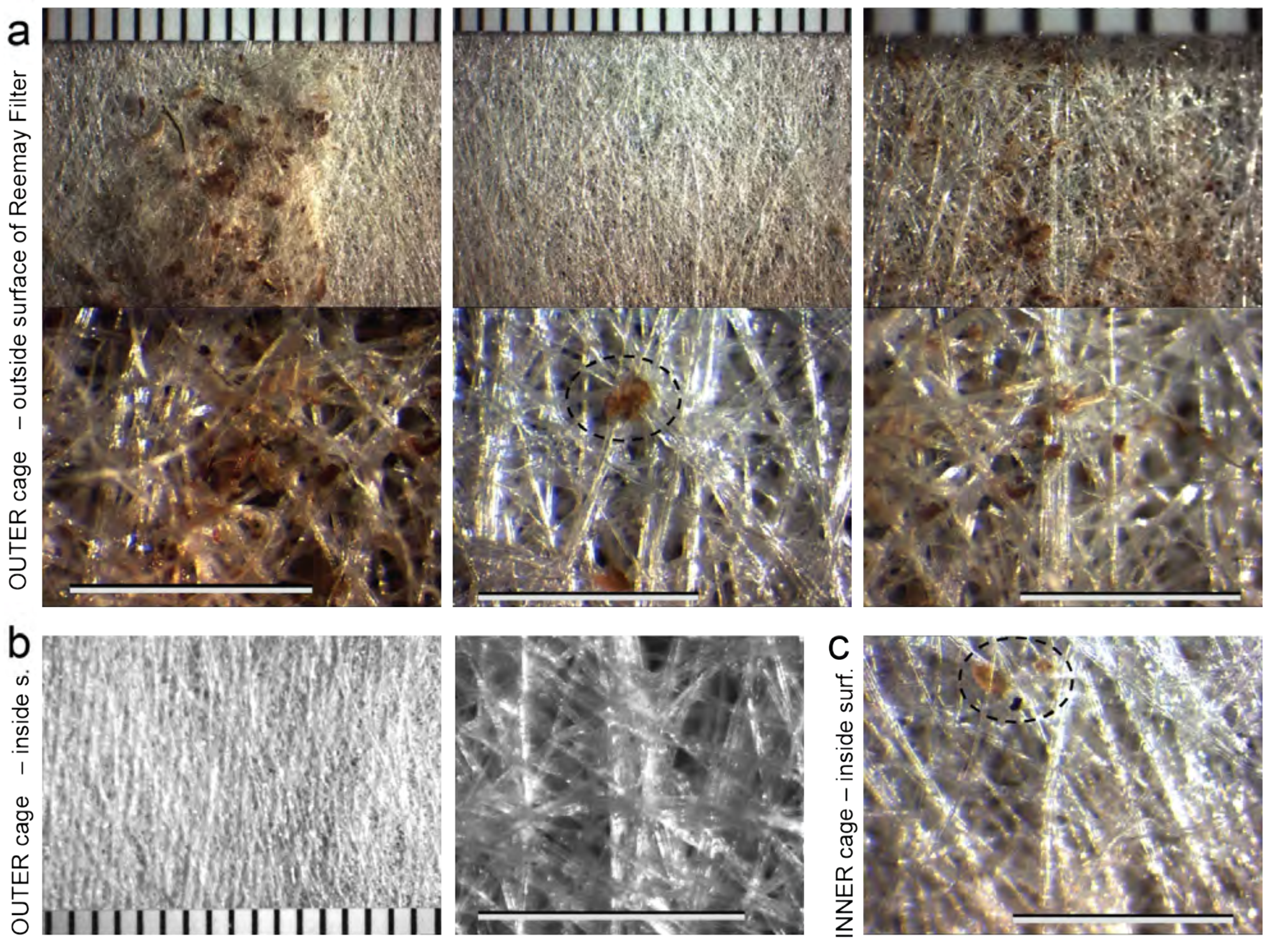

Supplementary Figure 3. Close-up photographs of the cage lid filters used in NesTiso.

a) Outer surface of the non-HEPA Reemay filter present in the lid of the outer cage in a NesTiso set left on a laboratory bench for 6 months. **b**) Clean inside (inner) surface of the same filter depicted in panel a. Abbreviation, inside s., inside surface. **c**) Soiled inside (inner) surface of the Reemay filter used in an inner cage of a NesTiso set that had housed GF SAMP mice that had been colonized with human fecal microbiota. Notice airborne particles at center of dashed oval. Scale bars, 1mm. Abbreviation, inside surf., inside surface.

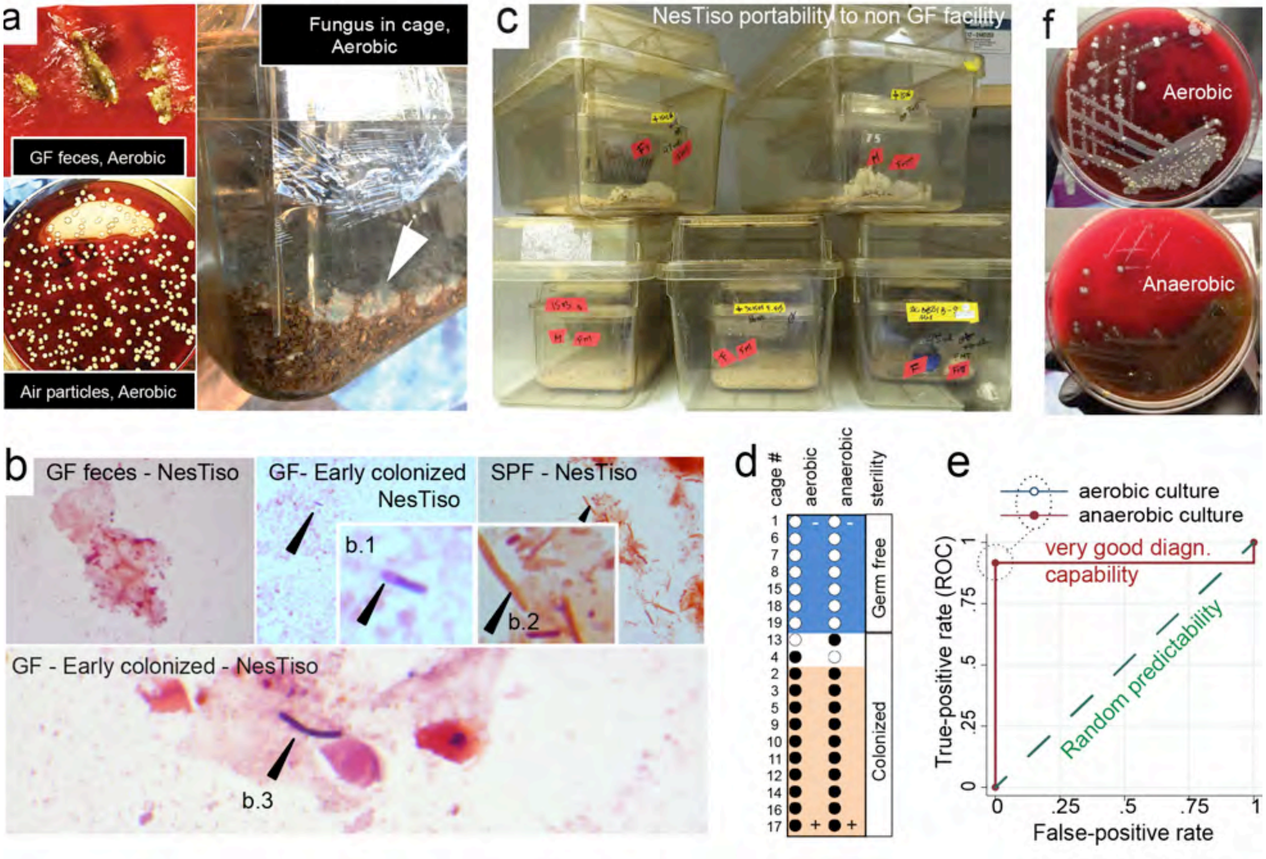

Supplementary Figure 4. Microbial Screening of GF mice and Portability of Nested Isolation Sets.

a) Culture examples on TSA agar. Top left: lack of bacterial growth in 'GF feces' of mice in NesTiso (37°C, 5d anaerobic). Bottom left: high density of bacterial colonies seeded by 'Air particles' in the room air of a BSL-2 research laboratory after an agar plate was left opened for 3h (37°C, 5d, aerobic; relevant for data in this figure panels d-f), Right: 'fungus in cage' ('fungal trap') on surface of moist soiled bedding of a NesTiso set colonized with environmental *Penicillium* spp. (incubated at 23°C, aerobic, 14d). **b**) Gram stain of fecal smears of representative GF and SPF mice. Notice the lack of bacteria in 'GF feces'. Notice distinguishable bacteria (morphology in a remarkable clean background; *i.e.,* no other microbes) in 'Early-colonized' GF-mice. Arrowheads; b.1, terminal-oval-deforming endospore-forming gram-positive rod (aerobic; *Bacillus* spp.); b.2, long gram-negative rod in SPF mice; and b.3, junction of two gram-positive dividing long-rod daughter cells in association with intestinal epithelial sloughed cells. Gram stain can detect bacteria in feces before quantitative changes in microbial DNA reach threshold for real-time PCR detection (estimated to be ~100 bacteria per 5-10 µg of fecal smear, if the mean number of *rrn* operons/bacterium was 10, and if one *rrn* operon provided one qPCR-detectable copy of DNA template). **c**) Photographic example of GF NesTiso sets that were mobilized out of the GF facility to a (non-GF) BSL-2 microbiological room, where feed-microbiota colonization experiments (panels d-f) were conducted. **d**) Culture test agreement between paired aerobic-and-anaerobic incubation of mouse feces after feeding thirty-two GF-mice in 19 NesTiso sets an irradiated non-GF diet (binary yes/no data). Aerobic incubation predicted a 10-day contamination outcome correctly in 18 of 19 cohorts. **e**) ROC analysis of data from panel d shows the predictability of aerobic incubation is similar to that of anaerobic incubation (ROC P=1.0). **f**) Paired fecal culture of feces from a mouse in TSA agar illustrates that bacterial growth is exuberant and colony differentiation more notorious under aerobic conditions (5d of incubation, 37°C).

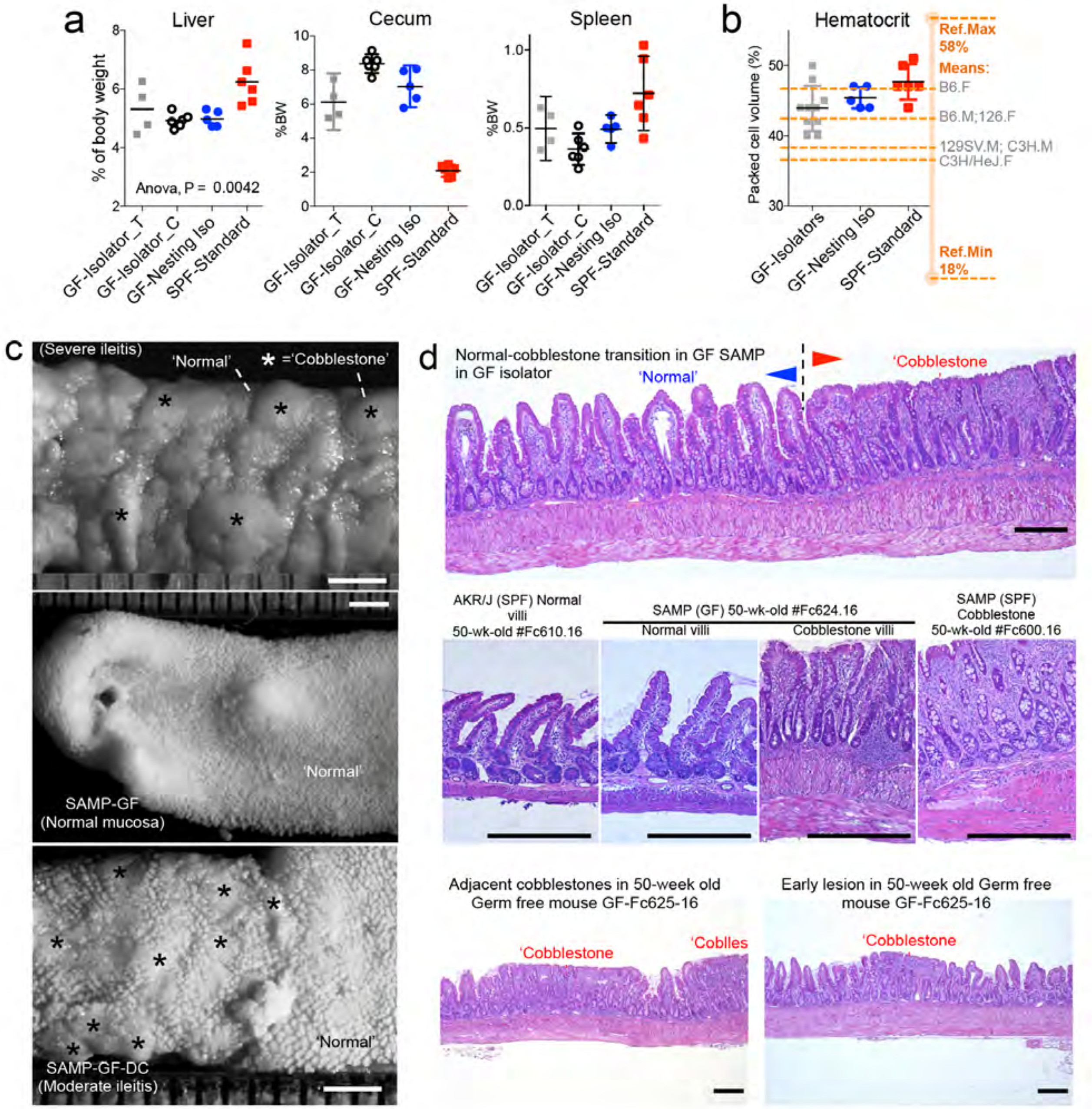

Supplementary Figure 5. Organ dimensions, hematocrit and ileitis phenotypes are not affected by NesTiso.

a) Univariate plots of data variability within the expected range for the SAMP mice tested. [1] Prospectively, an experiment assessing the effect of NesTiso in multiple organs (SPF and GF SAMP mice born and raised for 17 weeks either in isolators or NesTiso at CWRU, or isolators at Taconic Bioscience illustrates as expected that the normalized organ sizes and the hematocrit (packed red cell volume, surrogate for dehydration/erythrocythemia) are within expected variability and not affected by NesTiso. **b**) Hematocrit data and published reference intervals for various mouse strains[2-4] (mean±SD). **c**) Stereomicroscopic appearance of mild, moderate and severe cobblestone ileitis in SAMP mice to serve as reference to images shown in **Figure 2c**. 3-D-stereomicroscopic analysis of intestinal samples of 45-50-week-old mice confirms the presence of 3D-lesions typical of cobblestone ileitis in SAMP mice raised in GF-NesTiso. Scale bar, 1mm. **d**) Histological transition between 'cobblestone' lesion and normal mucosa in GF SAMP mice with ileitis in NesTiso. Scale bar, 250 µm.

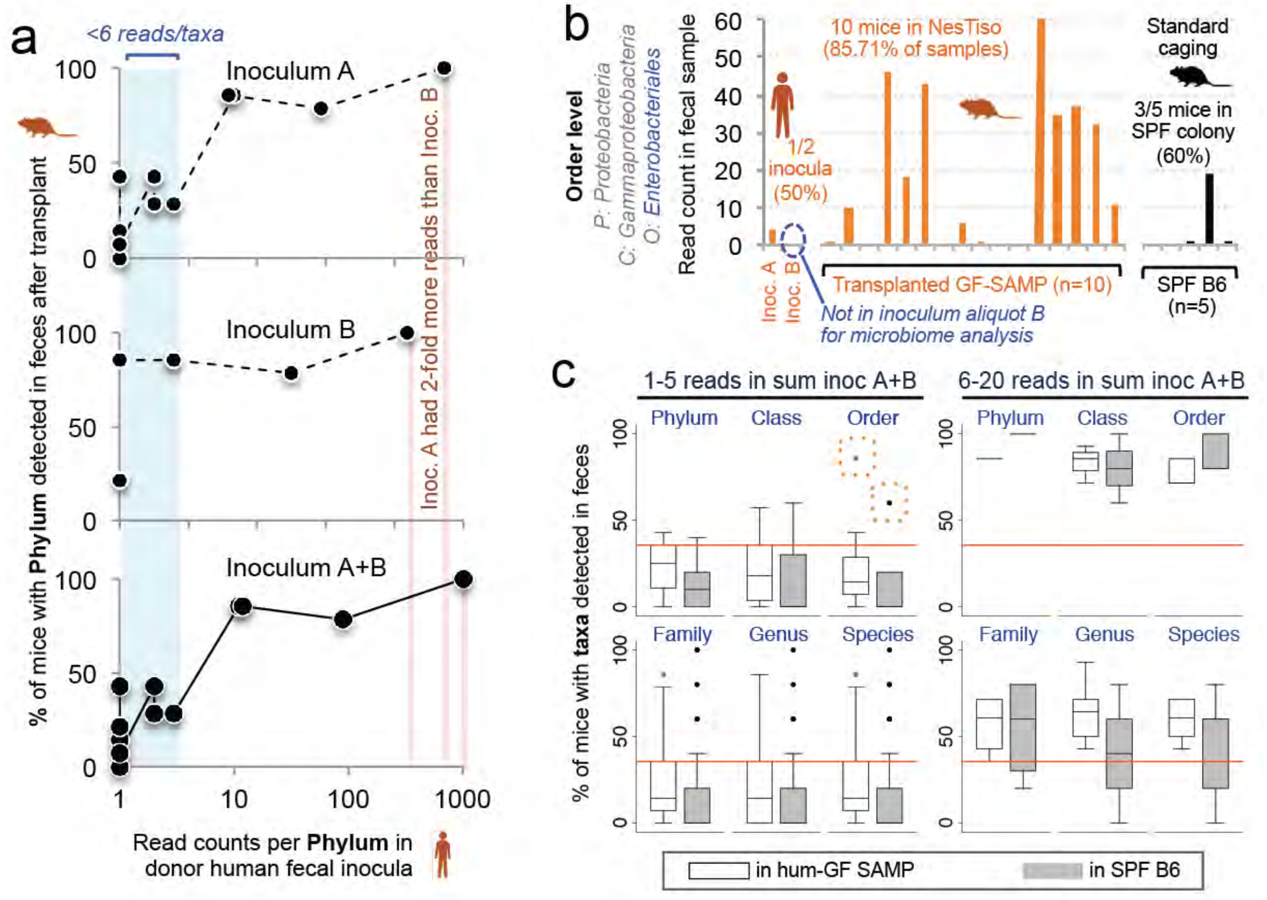

Supplementary Figure 6. Analysis of 16S rRNA microbiome recovery probabilities after microbiota transplantation is preferable to be conducted interpreting microbiome sequence reads in series without eliminating taxa/samples with low counts.

a) Correlation of read counts for all data at the Phylum level in this experiment for the human donor feces (inocula A and B), the sum of both (interpretation in series), and the percentage of mouse samples with taxa identified in their feces. **b**) Specific example at the order level for *Enterobacteriales*. Notice that both human inocula have either 4 or 0 reads (≤5 reads) per sample, but their isolation from the mice in GF-grade NesTiso showed that 85% of samples had been colonized by that taxa. From a binary statistics standpoint, having analyzed the mouse data in the context of inoculum B alone would have erroneously led to concluding that the taxa in mice was due to contamination. The confirmed GF status of mice in our GF NesTiso strategy, and the control of contamination events showed that cage-cage cross contamination is 100% unlikely. In this context NesTiso enables interpreting that the taxa present in the humanized mice are present due to the microbiota transplant and not due to cross contamination from other SPF mouse cages. **c**) Box plots represent the number of taxa with corresponding raw reads added (*i.e.,* interpreted in series) from both human inocula (A + B). Each outlier point represents unique taxa. Notice that removing data with 1-5 reads per taxa (e.g., OTU) will remove a unique bacterial order that was present in 80% of samples from GF mice transplanted with the human feces, and 60% of (3/5) B6 mice tested from our SPF facility (dotted areas). Reference lines represent higher value of higher boxplot in each panel.

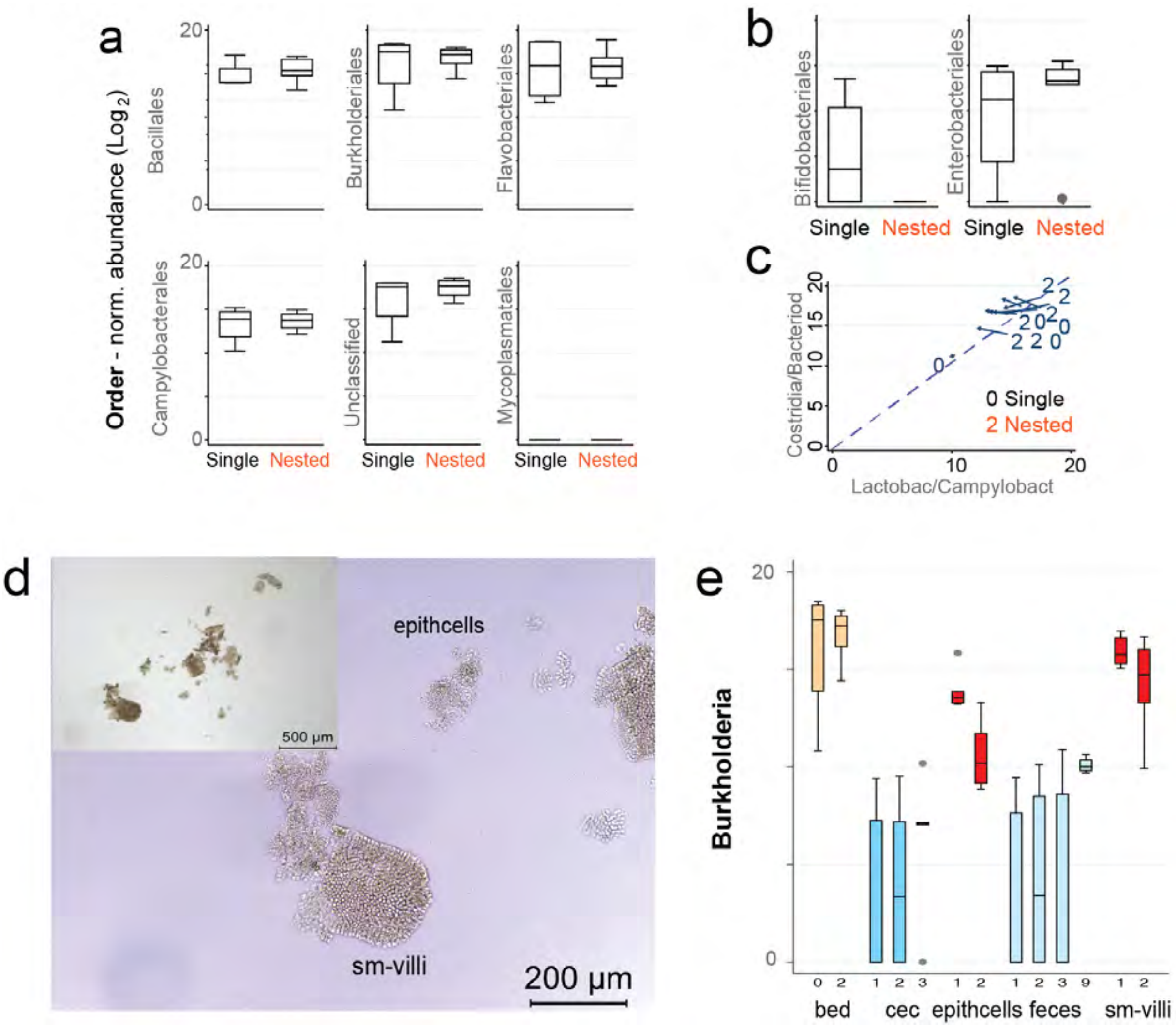

Supplementary Figure 7. Microbiome of bedding samples in the context of other samples also illustrates enrichment of *Burkholderiales*.

a) Additional representation of 16S rRNA microbiome bacterial orders that are similar in the bedding material after incubation in both NesTiso and Single standard static cages. **b**) *Bifidobacteriales* is the only example where there was differential effect due to caging, however, the discrepancy is in the context of cyclical bias likely irrelevant (and a false discovery) because *Bifidobacteriales* are mainly strict anaerobes and cannot proliferate in the bedding material outside the host. **c**) The microbiome of NesTiso and Single caging are the same after incubation. Paired correlation plot for four orders showed that the mean distances between the two plots are narrow and follow a linear prediction that intersects with zero further suggesting there are no differences in the fecal microbiome changes that can be attributed solely to the use of NesTiso. **d**) Photograph of epithelial cells and ileal villi sample data to contextualize the bedding microbiome analysis. **e**) 16S rRNA microbiome read abundance in bedding microbiome samples [single cages (0); NesTiso (2)] illustrating there is bedding enrichment of *Borkholderiales* with respect to fecal and cecum mouse samples. The parallel abundance of the same order in the epithelial cells suggests that the microbes in this *Borkholderiales* order may increase as cages get soiled and have biological relevance on digestive/animal phenotypes.

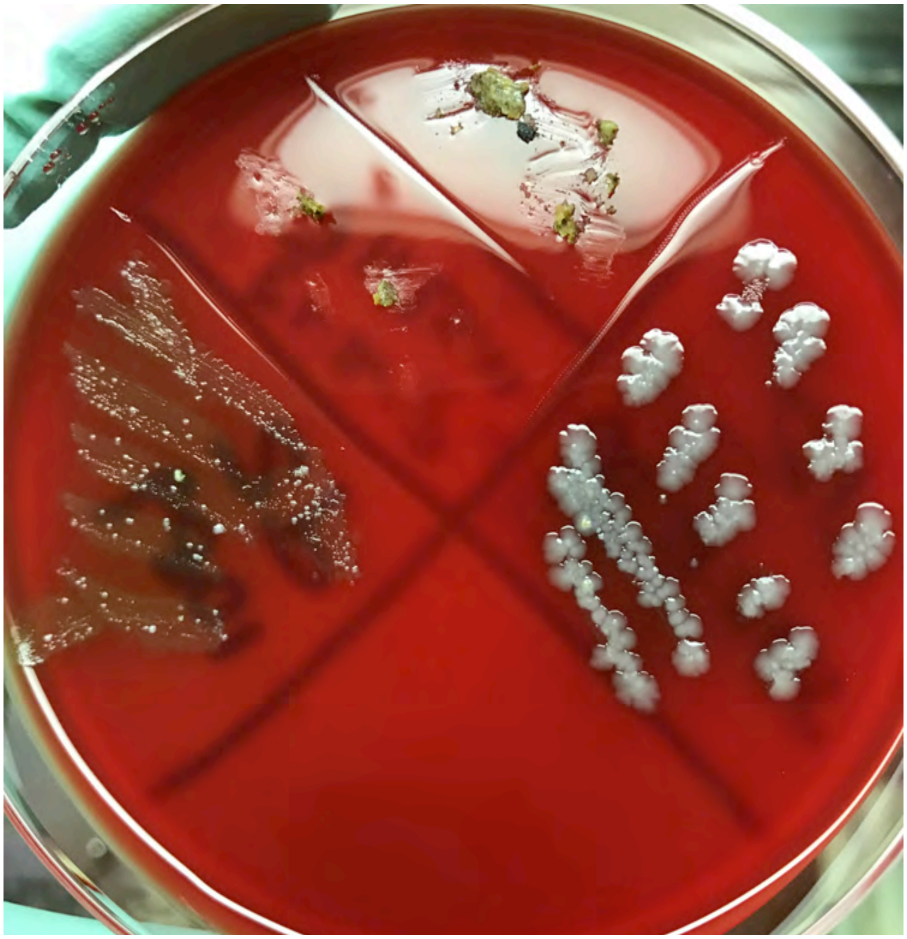

Supplementary Figure 8. Fecal samples illustrated on Figure 5a after 36h of incubation on Tryptic Soy Agar supplemented with 5% of defibrinated sheep blood. Left and right quadrants have feces from two littermate mice cohoused for > 20 weeks. Top panels have feces from two distinct GF mice (no bacterial growth). Bottom quadrant was not inoculated (negative control). Notice the two cohoused mice have a very distinctive cultivable phenotype. This co-streaking assay was used to monitor the effect of bedding soiledness on intestinal cyclical microbial bias (see text, Figures 6-7 and Supplementary Fig. 10).

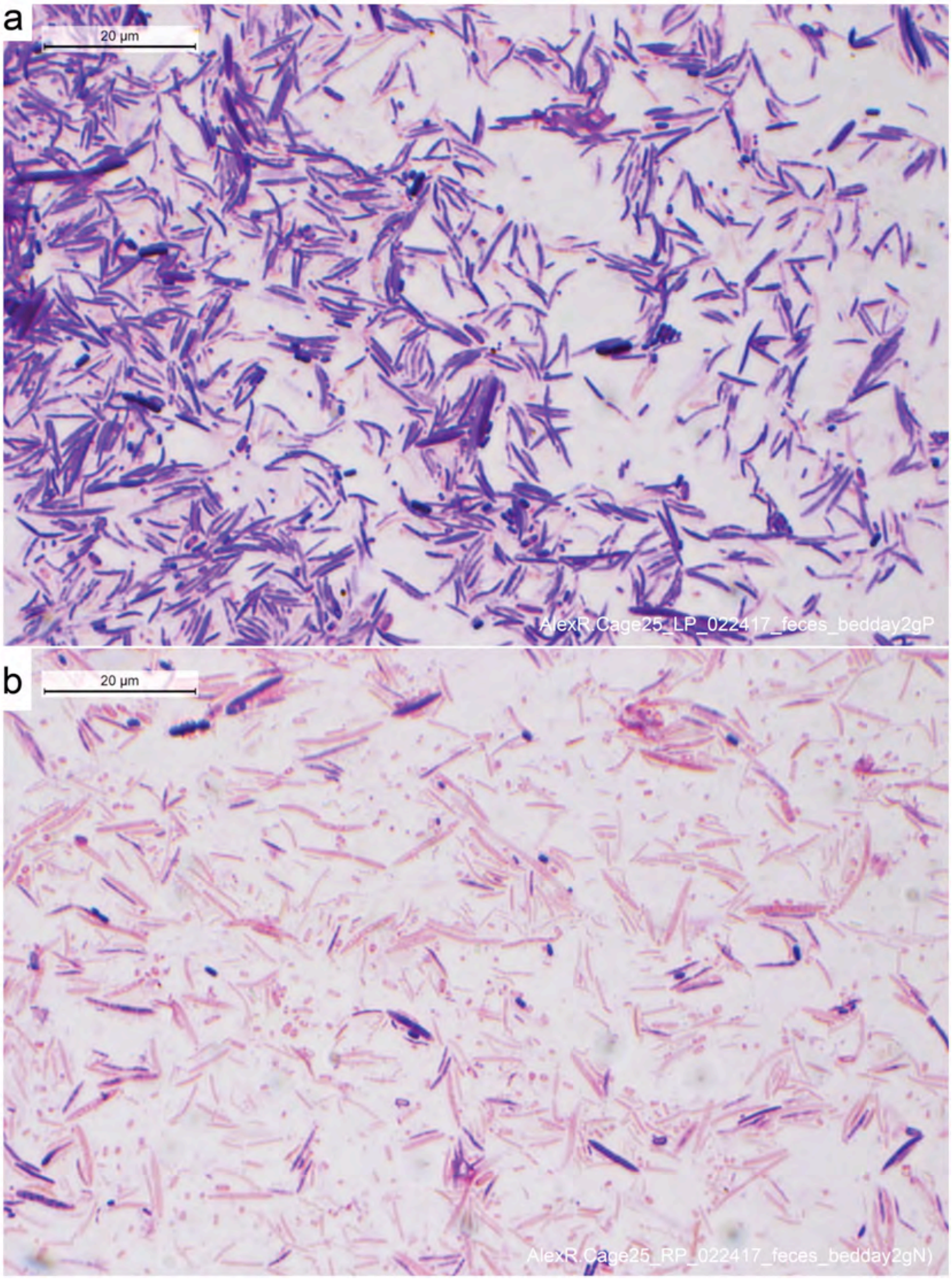

Supplementary Figure 9. Gram stain of fecal sample illustrates distinct cohoused gut microbiome profiles are long lasting. a) Mouse with relative higher gram-positive:gram-negatve microbial ratio. **b**) Mouse with relative lower gram-positive:gram-negatve microbial ratio, indicates there is more gram negative organisms and possible LPS-associated antigenic exposure. Images correspond to the same 5 cohoused mice cage#25 depicted in **Figure 5a** three months earlier, notice that the co-streaking culture assay profile have also distinct correspondent fecal profiles based on gram-stain of fecal smear used to streak the agar.

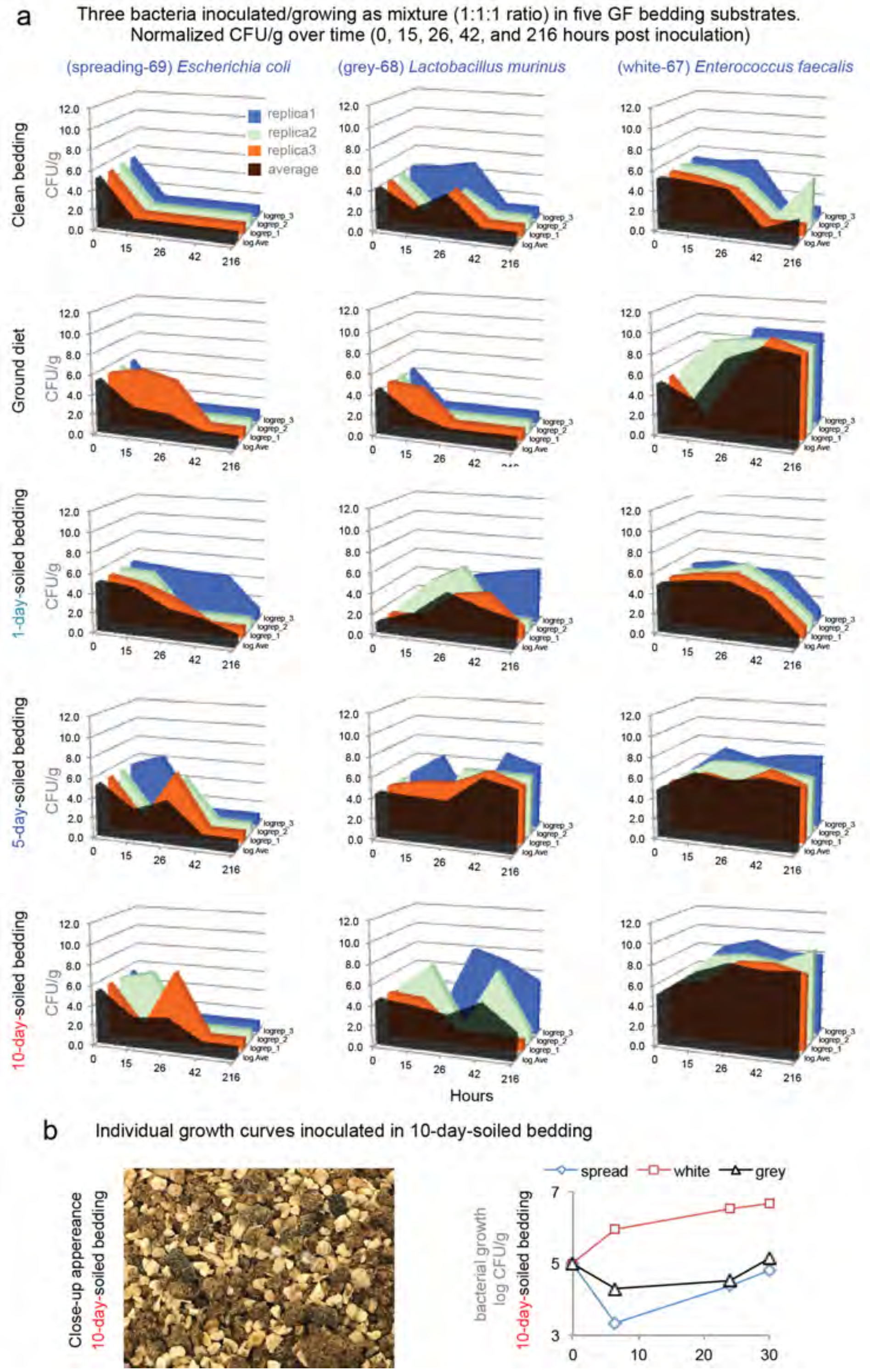

Supplementary Figure 10. Growth and survival of three abundant fecal murine bacteria incubated in various (corncob bedding:diet) substrates. a) Competition split plot experiment where 3 bacteria were inoculated as a cocktail mixture 1:1:1. Notice *Enterococcus faecalis* is the most suitable [2] to grow in all conditions, and remains viable after 9 days of incubation despite dehydration of substrates. *Escherichia coli* in contrast is the least suited to grow in bedding. Notice inhibitory effect of diet in *L. murinus* and *E. coli*. (CFU/g data are shown in Log_{10} units). **b**) Individual 30-hour growth curves confirm that *Enterococcus faecalis* (white) is the fastest growing organism in soiled bedding. See *Lactobacillus murinus* grows better on 5-day-soiled bedding in **Figure 6**.

Supplementary Table 1. Pathogens Tested by Serology in Germ-Free Mice in Nested Isolation.

Pathogen	Disease Description	Transmission
Bacterial CARB	Chronic pneumonia. Cilia-associated respiratory bacillus - bacterial pathogen. Gram-negative, non-spore-forming. Undefined (*Flexibacter*, *Fusobacterium*).	Primarily via direct contact, often within 1st week of birth. Bedding sentinels not efficient means for detection. *Bordetella avium, M pulmonis,* differential diagnoses.
Clostridium piliforme (Zoonotic)	Tyzzer's disease. Asymptomatic; necrotizing hepatitis, ileitis, typhlitis, or colitis.	Gram-negative filamentous rod-shaped spore-forming bacterium. Ingestion of spores in environment or feces. Spores infectious.
Mycoplasma pulmonis	Murine mycoplasmosis. Chronic suppurative bronchopneumonia, lymphoid hyperplasia.	Uterus, middle ear, joints also affected. Direct contact, aerosol; transplacental transmission.
Fungal		
Encephalitozoon cuniculi (Zoonotic)	*Mycrosporidial* (fungal) parasite; rabbits, rodents. Obligate intracellular eukaryotic parasite. Gram-positive.	Renal, neurologic or ocular disease. Ingestion of spores in urine, inhalation. Vertical transmission.
Viral		
Ectromelia virus (Zoonotic)	DNA enveloped. Murine Poxvirus. Family Poxviridae, genus Orthopoxvirus.	Virus found in scabs and feces for >16 weeks post-infection. Exposure via cutaneous trauma. Many routes of infection, direct contact or by fomites.
EDIM	RNA non-enveloped. Epizootic diarrhea of infant mice virus, murine rotavirus.	Shed in large amounts in feces, fecal oral transmission. No vertical transmission.
Hantaan virus (Zoonotic)	RNA enveloped. Murine hantavirus. Asymptomatic. Cell lines. Serious infection in humans.	Shed persistently in feces, urine, saliva. Transmission by direct contact or contact with urine or feces. Vertical transmission unlikely.
K Polyoma virus	DNA non-enveloped. Mouse pneumotropic virus. Polyomavirus. Cell lines.	Interstitial pneumonia, lytic lesions, tumors. Ingestion of contaminated feces, or inhalation. Persistent infection, at any age.
LCMV (Zoonotic)	RNA enveloped. Lymphocytic choriomeningitis virus (zoonotic murine arenavirus). Asymptomatic. Lymphocytic infiltrates in liver, adrenal, kidney, lung). Cell lines.	Contact with saliva, nasal secretions, or urine. Vertical transmission. Virus infects female germ cells, sperm. Immune-complex glomerulonephritis, and vasculitis. Inhibits tumor formation by other viruses.
LDV	RNA enveloped*. Lactate dehydrogenase-elevating virus. (murine arterivirus). Paralytic syndrome in AKR/J.	Persistently viremia after infection. Contact via bite wounds or sexual contact. Vertical, transplacental or via milk.
MAV1 (FL)	DNA non-enveloped. Murine adenovirus, strain 1 (FL). Asymptomatic.	Transmitted via direct contact with urine, feces, nasal secretions. Type A intranuclear inclusions in adrenal gland.
MAV2 (K87)	DNA non-enveloped. Murine adenovirus, strain 2 (K87). Asymptomatic.	Transmitted via direct contact with feces. Type A intranuclear inclusions ileum/cecum.
MCMV	DNA enveloped. Herpesvirus. Murine Cytomegalovirus /muromegalovirus. Asymptomatic. CNS and myocardium.	Excreted in tears, saliva, urine. Vertical transmission may occur. Natural infections localized to salivary glands.
MHV	RNA enveloped. Mouse hepatitis virus. Betacoronavirus. Cell/tumor lines.	Asymptomatic. Respiratory, enterotropic, polyotropic strains. Highly contagious in aerosols, fomites, and contact with feces.
MNV	RNA non-enveloped. Murine norovirus. Caliciviridae. Asymptomatic, fecal shed.	Transmitted via fecal-oral route. Hepatitis, peritonitis, and interstitial pneumonia. Replication in macrophages.
MPV	DNA non-enveloped. Mouse parvovirus 1-5 and NS1. Asymptomatic.	Direct contact. Shed in urine, feces, oronasal secretions. Resistant in dust. **
MVM (MMV)	DNA non-enveloped. Minute virus of mice, murine parvovirus. Asymptomatic.	More pathogenic for hematopoietic cells than mouse parvoviruses. **
MTV	Mouse thymic virus, murine herpesvirus	
PVM	RNA enveloped. Pneumonia virus of mice, paramyxovirus. Also non-suppurative vasculitis	Transmitted by aerosol and direct contact with respiratory secretions. **
REO3	RNA non-enveloped. Asymptomatic. Murine Reovirus. Stunting, diarrhea, encephalitis.	Virus shed in feces. Fecal-oral transmission. Direct exposure to airborne dust. No vertical transmission.
SEND	RNA enveloped. Sendai, respirovirus. Paramyxoviridae. Pneumonia, dyspnea. Fatal.	Aerosol and respiratory secretions. Not transmitted by bedding.
TMEV	RNA enveloped. Theiler's murine encephalomyelitis virus. Cardiovirus str. GDVII.	Transmitted via fecal-oral route. Asymptomatic, paralysis or fatal.

Mouse ID number, sex and ages of six tested animals were from 5 separate N2LI cohorts, DC-1506A (M, 29.43 week-old), DC-Br1502P (F, 46.43), DC-Br1502P (M, 42.43), DC-1505A (F, 59.43), DC-Br1503A (F, 60.43), and DC1506 (M, 67.43).

*PCR and serology is advised for virus confirmation.

**Prevention is documented by using cage filter lids. [5,6] Studies on concurrent pathogen prevalence in laboratory rodents from >500 centers in North America, Europe and Asia (over half million mice and rats), have illustrated the prevalence of commonly detected pathogens in mouse rearing facilities that have an impact on animal phenotypes or have zoonotic potential. [7-14]

Supplementary Table 2. Two-year estimated contamination incidence of GF mice in Nested Isolation based on cage replacements every 10 days and two mouse colony inventory snapshots.

	Cage counts [a] Animal density			Age [b] (weeks)		Estimated contamination incidence [cd] (mouse-, cage-days, cage-openings)				
**Mouse colony inventory**	**cage count**	**adult (pups)**	**mice/cage**	**Adult mice mean ± SD**	**Oldest GF mouse**	**Contamt. cages Cumulative, n=**	**Cage-days**	**Mouse-days**	**Mouse-days per cage**	**Cage openings**
8 month inventory snapshot										
B-Room 1 - Isolator 1	4	11(8)	4.8	25.4±6.8	38	-	2,920	13,870	3,467	292
B-Room 1 - Isolator 2	2	5(8)	6.5	22.4±2.2	24	-	1,460	9,490	4,745	146
Total Pressurized Isolator	*6*	*16*	*2.6*	*23.9*	*38*	0	*4,380*	*23,360*	*3,893*	*438*
B-Room 1 - Nested Isolation	6	13	2.2	23.8±15.4	45	1	4,380	9,490	1,581	438
E-Room 2 - Nested Isolation	7	13	1.9	26.9±1.8	29	-	5,110	9,490	1,355	511
Total NesTiso sets	*13*	*26*	*2.0*	*25.4*	*45*	*1*	*9,490*	*18,980*	*1,460*	*949*
22 month inventory snapshot										
B-Room 1 - Isolator 1	4	11(5)	4.0	30.6±5.8	45	-	2,920	11,680	2,920	292
B-Room 1 - Isolator 2	4	10(5)	3.8	25.2±6.0	34	-	2,920	10,950	2,738	292
B-Room 1 - Isolator 3	3	8	2.7	23.9±3.7	33	-	2,190	5,840	1,947	219
B-Room 2 - Isolator 4	4	11(10)	5.3	25.5±3.7	49	-	2,920	15,330	3,833	292
Total Pressurized Isolator	*15*	*40*	*2.7*	*26.3*	*49*	0	*10,950*	*43,800*	*2,920*	*1,095*
B-Room 1 - Nested Isolation	28	65(11)	2,7	24.9±16.2	52	+1	20,440	55,480	1,981	2,440
E-Room 2 - Nested Isolation	21	47	2.2	29.9±13.6	45	-	15,330	34,310	1,633	1,533
Total NesTiso sets	*49*	*112*	*2.3*	*27.4*	*52*	*2*	*35,770*	*81,760*	*1,807*	*3,973*
Linear colony growth cumulative adjusted estimates for 2-y study AUC [e]	***64***	**-**	***2.7***	**-**	**-**	***2***	***23,360***	***62,780***	***981***	***2,534***

This Supplementary table is an expansion of **Table 1** for comparative purposes of the two inventory snapshots as the number of animals and cages grew steadily over the life of the study.

[a,b] Isolators housed single static cages with young, active, or retired breeders ≤3) and pups. NesTiso cages were mostly used for nonbreeding mice. Totals (averages) for animal density and ages are based on adult mice data (no pups) to illustrate comparability of breeders with nonbreeding mice. Note that age in Nested isolation and Isolators are comparable.

[c] mouse-days or cage-days=n of mice or cages x 730 days; mouse-days/cage = mouse-days ÷ n of cages; cage openings = cage-days ÷ days interval between cage replacement. The two contaminated cages occurred on two separate months of the study.

[d] Inventory snapshots of mouse colony in this experiment at months 8 and 22 were used for crude estimations for a 2-year period, assuming a constant number of mice and cages. Crude estimations of more realistic estimates were derived assuming a *linear growth of the colony reflecting an increment of the cage count of 2 cages per month, for a colony* expansion from 1 to 49 cages for months 0 to 22.

[e] A geometric estimating approach based on area under the curve is as follows: 64 cages x 2.74 mice/cage/day x 730 days x 0.5 AUC = 64,006 mouse days, which is similar to the 62,780 reported in the table. These approximations are conservative underestimating the actual efficiency of NesTiso in preventing new contaminations, and cage-cage contaminations, since we have at times housed larger number of cages in the study rooms.

Supplementary References

1 Rodriguez-Palacios, A. *et al.* Stereomicroscopic 3D-pattern profiling of murine and human intestinal inflammation reveals unique structural phenotypes. *Nat Commun* **6**, 7577, doi:10.1038/ncomms8577 (2015).

2 Russell, E. S., Neufeld, E. F. & Higgins, C. T. Comparison of normal blood picture of young adults from 18 inbred strains of mice. *Proc Soc Exp Biol Med* **78**, 761-766 (1951).

3 Russell, E. S. & Bernstein, S. E. Blood and Blood Formation. In: Biology of the Laboratory Mice. http://www.informatics.jax.org/greenbook/frames/frame17.shtml (ed E L. Green). Chapter 17 (1966).

4 Schlager, G. & Weibust, R. S. Selection for hematocrit percent in the house mouse. *J Hered* **67**, 295-299 (1976).

5 Brielmeier, M. *et al.* Microbiological monitoring of laboratory mice and biocontainment in individually ventilated cages: a field study. *Lab Anim* **40**, 247-260, doi:10.1258/002367706777611497 (2006).

6 Lipman, N. S., Corning, B. F. & Saifuddin, M. Evaluation of isolator caging systems for protection of mice against challenge with mouse hepatitis virus. *Lab Anim* **27**, 134-140, doi:10.1258/002367793780810360 (1993).

7 Liang, C. T. *et al.* Microbial contaminations of laboratory mice and rats in Taiwan from 2004 to 2007. *J Am Assoc Lab Anim Sci* **48**, 381-386 (2009).

8 E, J., Jiang, Y. T., Yan, P. F. & Liang, J. P. Biological changes of Enterococcus faecalis in the viable but nonculturable state. *Genet Mol Res* **14**, 14790-14801, doi:10.4238/2015.November.18.44 (2015).

9 Mähler, M. & Köhl, W. A serological survey to evaluate contemporary prevalence of viral agents and Mycoplasma pulmonis in laboratory mice and rats in western Europe. *Lab Anim (NY)* **38**, 161-165, doi:10.1038/laban0509-161 (2009).

10 Baker, D. G. Natural pathogens of laboratory mice, rats, and rabbits and their effects on research. *Clin Microbiol Rev* **11**, 231-266 (1998).

11 Pritchett-Corning, K. R., Cosentino, J. & Clifford, C. B. Contemporary prevalence of infectious agents in laboratory mice and rats. *Lab Anim* **43**, 165-173, doi:10.1258/la.2008.008009 (2009).

12 Manjunath, S. *et al.* Sero-Prevalence of Rodent Pathogens in India. *PLoS One* **10**, e0131706, doi:10.1371/journal.pone.0131706 (2015).

13 Oliveira Moreira, J. C. *et al.* Certification of microbiological diagnostic methods of the CEMIB/UNICAMP sanitary quality control laboratory together with ICLAS [Certificação de métodos microbiológicos de diagnóstico do Laboratório de Controle Qualidade Sanitária do CEMIB/UNICAMP junto ao ICLAS]. *Revista Saberes Universitarios* **1**, 50-62 (2016).

14 Knust, B. *et al.* Lymphocytic choriomeningitis virus in employees and mice at multipremises feeder-rodent operation, United States, 2012. *Emerg Infect Dis* **20**, 240-247, doi:10.3201/eid2002.130860 (2014).

“Cyclical Bias” in Microbiome Research Revealed by A Portable Germ-Free Housing System Using Nested Isolation

Alexander Rodriguez-Palacios, Natalia Aladyshkina, Jessica C. Ezeji, Hailey L. Erkkila, Mathew Conger, John Ward, Joshua Webster, and Fabio Cominelli; Case Western Reserve University School of Medicine, Cleveland, OH 44106, USA. axr503@case.edu

Mathematical Visualization of Cyclical Bedding-Dependent Microbial Growth and Cumulative Selection Bias: Fast vs. Slow Growers.

This supplementary appendix illustrates the supporting rationale of **Figure 4g** presented in the research article entitled: **“Cyclical Bias in Microbiome Research Revealed by A Portable Germ-Free Housing System Using Nested Isolation”** by Alexander Rodriguez-Palacios, *et al* (*Scientific Reports*, 2018, https://doi.org/10.1038/s41598-018-20742-1). In mathematics, a periodic function is a function that repeats its values at regular intervals or periods, which can be used to describe oscillations, waves, and other undulating cyclic phenomena. Here, we illustrate the theoretical periodicity dynamics that could be observed among some microbial members of the bedding microbiota due to regular replacement of the bedding material. We used validated logistic modelling with event-based cycle functions to visualize the potential outcomes of microbiota bias (depletion or enrichment) in the bedding material of mouse cages. We highlight biological assumption rules that may have an impact on mouse microbiome research.

1. Rationale for Mechanistic Visualization of Microbial Dynamics.

In epidemiology, mathematical models have two distinct roles. Predictive roles and mechanistic roles. Predictive modelling of future dynamic behaviors require high accuracy and parameters relevant to the biological processes to be predicted. Mechanistic modelling are designed to allow the understanding of dynamic interactions in a theoretical context in which precise biological data is not necessary to enable the performance of the model or the study of biologically plausible and implausible possibilities. Here, we are relying on the mechanistic possibility of modeling as we seek to understand through visualization the pattern of microbial selection (bias) that we observed for the enrichment of several bacterial orders in the microbiome data presented in **Manuscript Figure 4g**. The precise prediction of the microbiome changes over time are considered an optional more detailed expansion of the principles that are herein described. Because obtaining data for precise predictions is subject to at least 5 factors (herein referred to as Periodicity Rules, see below), future predictive models would require additional data which will be highly dependant on the factors listed.

2. deSolve R Package

Herein, we used a previously validated model system. As a mechanistic approximation of bacterial growth visualization the model has been widely used to describe microbial growth in liquid media. We implement such principle using logistic modeling and the introduction of growth breaks (new cage replacements) using recently developed ‘event’ function equations in open-source *deSolve* package (v1.20, July 14, 2017). deSolve is a compilation package that solves initial value problems of differential equations (‘ODE’, ‘DAE’, ‘DDE’) that was developed by Karline Soetaert, *et al.* in R software.

3. Biological Assumptions

The bedding material in the cages of experimental mice are constantly enriched with the organic content of mouse-derived diet, feces, urine and other bodily secretions, and moisturized by urine, perspiration, respiration, and the spillage of drinking water. For the purpose of this module, we assume that the integration of organic solid elements/nutrients (mainly, feces and pelleted food) with the rest of the bedding material is homogeneous as it would be the case for dissolvable nutrients in liquid media. From a microbiological standpoint, at time 0, when a set of mice are transferred to a new clean cage, the seeding bacteria is never zero at the beginning of each cage cycle as bacteria are always present in feces. Due to the solid nature of feces, herein we assume for modeling simplicity, that the microbes are spatially confined within the fecal pellet and clustered among the surrounding elements of the bedding material.

Herein, we assume that the addition of moisture, and food nutrients makes the bedding material a nutritious environment that could favor some microorganisms and not others. To visualize the effect that adding new bedding to the cage has on population dynamics, we use a validated logistic growth method that has been validated for liquid nutritious conditions of bacterial growth were new fresh media is added to a spatial unit as bacteria reach maximum growth capacity. The model, partly described in the deSolve R package, is deterministic (no random variables included) and discrete (because it models organisms, and not fractions of it).

4. Logistic Model of Bacterial Growth in a Single Batch: Fast-Slow Growers

A logistic regression model describes the dynamics of bacterial growth providing an s-shape curve. In the illustration below, the bottom flat segment of the line at the start in the left side of each panel represents the lag phase where small number of bacterial at a rate whose changes in absolute counts or optic turbidity of the substrate (media) are not perceptible. The lag phase is gradually followed over time by the steep slope observed towards the middle of the curve representing the linear exponential (also called logarithmic) phase of bacterial growth, where depending on the condition each bacterial cell divides resulting to two new cells (the mother cell and its actively metabolic daughter cell). Accounting for the doubling time (speed of cell division) of the population, the panels below in **Illustration 1** show three curves having three different growth rates (r=0.05, 0.1 and 0.9).

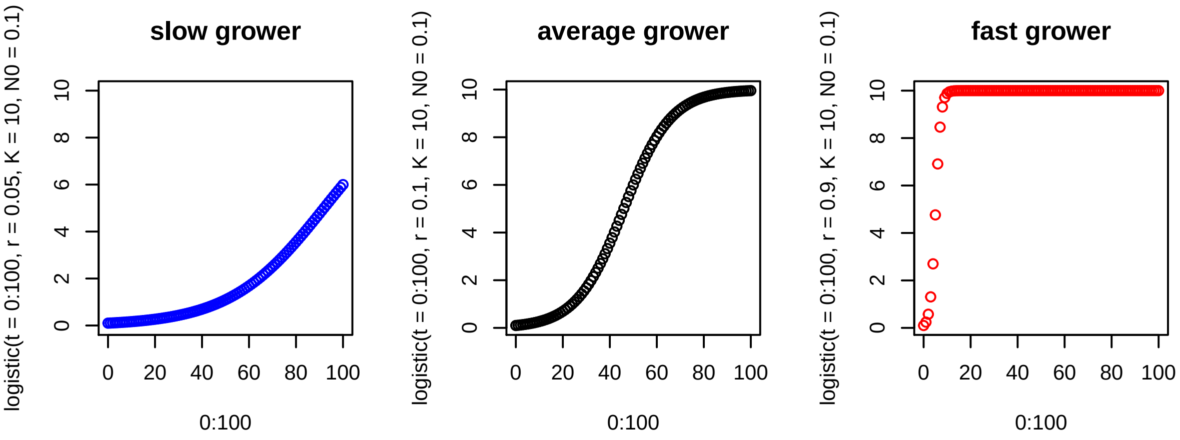

Illustration 1. ***Logistic model of bacterial growth with different growth rates.*** The logistic model depicted is $dN/dt = r * N * (1 - N/K)$ for the which the analytical solution $Nt = K * N0 * exp(r * t)/(K + N0 * (exp(r * t) - 1)$ as described in *deSolve*.

5. Linear Accumulation of Fecal Pellet Over a 10-day Cage Cycle

At the beginning of each clean break, the number of animals would determine the number of fecal pellets that would seed the bedding material with bacteria. We assumed that bacteria would growth on the bedding material favored (growth rate increased) over time by the accumulation of fecal organic matter, which in turn increases linearly as a function of the number of mice housed in the cage and the length of time the mice are housed. Here we assumed that the production of feces by each mouse is constant (**Illustration 2**), which we validated experimentally with mice monitored over time.

```
## Warning: package 'deSolve' was built under R version 3.4.1
```

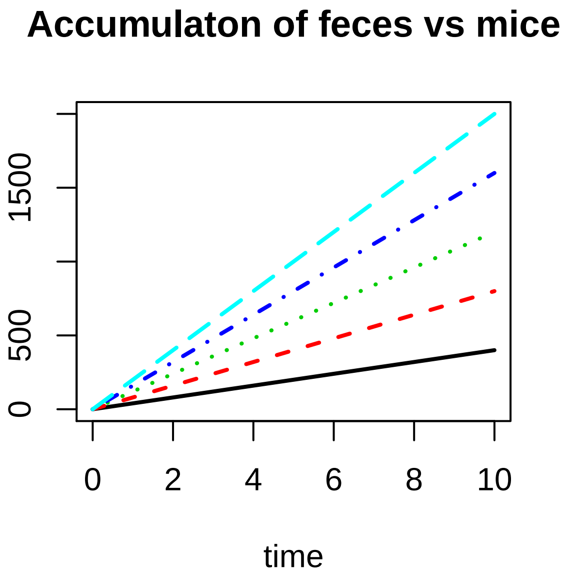

Illustration 2. ***Accumulation of fecal microbial biomass in cage bedding.*** The plot illustrates the linear correlation between the number of mice and expected number of acculated fecal pellets. From left to right, each line represents 5, 4, 3, 2 and 1 mouse projections.

6. Initial Fecal Microbial Seeding on Logistic Dynamics - A Cage Cycle

Here we used deSolve to find the numerical solution to the logistic functions since numerical solutions allow discrete forcings, events, such as the case of including time frames where bedding will be completely removed and replaced with new bedding (microbiome content becomes zero when cages are replaced, *i.e.*, time 0). We assume that bacterial decay dynamics is not a limiting factor for the purpose of this example, because the organic content in bedding is constantly increasing between cage replacements by the constant addition of organic matter from feces, urine, and ground diet particles. In this context bacterial growth is steady since there will be no growth restriction in the cycle because the medium (the soiled bedding) is constantly enriched. Assuming that a mouse defecates 40 fecal pellets per day and that the bacterial biomass in each pellet doubles every 24 hours (t, time step in graph, conservative estimate), each condition would have different microbial colonization (growth) rates. Assuming that the bedding material was saturated and there was no more nutrients for biomass doubling, bacterial growth for the pellets defecated by 5 mice over a 10 d course would not double, as they would reach the maximum carrying capacity of the bedding. **Illustration 3** depicts the effect of different amounts of fecal seeding biomass on the expected curves assuming that the cage had a maximum carrying capacity (as it is visualized in **Illustration 4** as a function of substrate). However, it is important to highlight that the amount of organic nutrients added to the floor accumulate as bedding at a constant rate (presumably) irrespective of the bedding conditions.

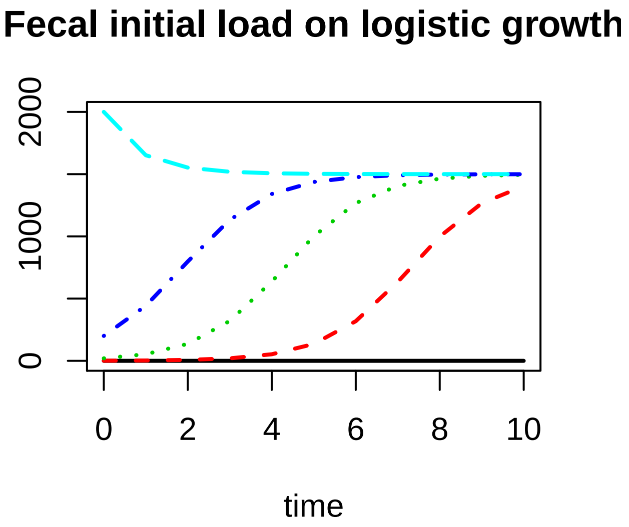

Illustration 3. ***Effect of fecal microbial biomass at beginning of cage cycle.*** The curves illustrate the growth dynamics of 5 seeding conditions (initial condition for number of fecal pellets seeded in bedding). From left/top to right/bottom curves: 2000 pellets, equivalent to 5 mice housed for 10 days; 100 pellets (5 mice for 1 day), 20 pellets (one mouse defecating only half the expected 40 pellets), 1 pellet, and 0 pellets (flat line, no biomass seeded, flat line at $y0$),

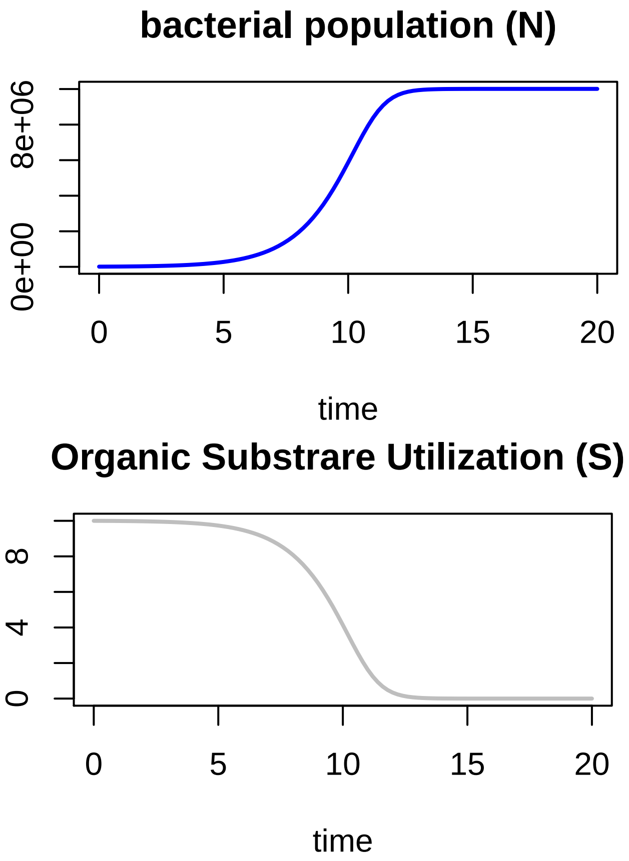

Illustration 4. ***Logistic Model of Substarte Utilization (S) and Bacterial Growth (N) in a closed culture batch.***

7. Event Functions: Reset Dilutional Effects with New Bedding Changes

To conceptualize and visualize the effect of replacing cages every 10 days, here we forced periodicity by implementing an event based function. The R code provided below, allows the resetting of the bacterial concentration in Bedding to zero, although it is not biologically zero since mice defecate and start the microbial seeding as illustrated above. On the other hand, the forced periodic events (user defined user) model the substrate as a rich source of nutrients that are at reset at time 0. **Illustration 5** shows the predicted fate of fast and slow growing microbes over several cage cycles.

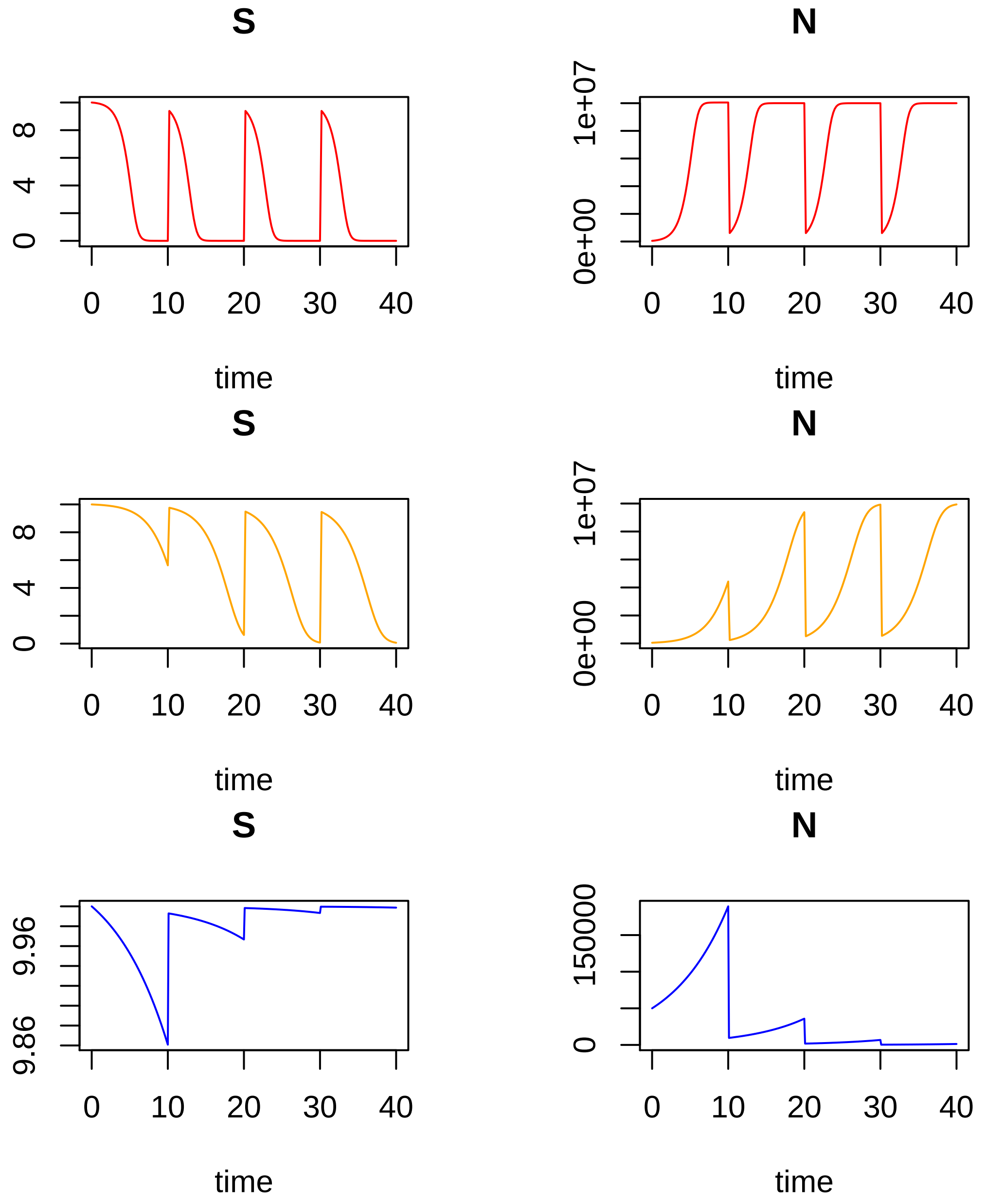

Illustration 5. ***Fast vs. Slow Growers.*** Slow growers could not outcompete other faster-growing microbes over time. Analysis predict that slow environmental microorganisms not suitable to survive in the mouse would become extinct over time and cycles. In this context, either form of outcompetition or extinction implies a functional source of microbial bias. S, nutrients; N, bacterial counts.

8. Periodicity Rules and Factors influencing intra-cage cyclical dynamics

Rule	Description
1	Not all bacterial species will grow in bedding.
2	Nutritious content of bedding material increases over time.
3	The number of mice in cage determines organic enrichment and moisture in the bedding.
4	The temperature of bedding material increases with the number of mice.
5	Each cage replacement cycle functions as a clean break.
6	High frequency of clean breaks can lead to extinction exclusively environmental microbes.

8.1 Considerations

Rule 1. Not all bacterial species will grow in bedding.

Bedding enriches aerotolerant, mesophilic at room temperature, do not require interaction with the host, and may survive in harsh conditions (dry bedding at beginning of cycle and low nutrient availability).

Rule 2. Nutritious content of bedding material increases over time.

At the time new bedding is added, the water microbial and nutritional content of the bedding material is very low. As animals defecate, eat and drink in the cage, the content of water (from leakage from bottle water, or urination), and organic matter (urine, feces, skin cells desquamation, other bodily secretions) increases.

Rule 3. The number of mice in the cage determines organic enrichment and moisture in bedding material.

As the number of animals increase, or the biomass and metabolic rate of the animal increases, the organic and moisture content increases.

Rule 4. The temperature of bedding material increases with the number of mice.

Thermal infrared studies indicate that the bedding material can be higher than the room temperature as animals irradiate heat within the cage.

Rule 5. Each cage replacement cycle functions as a clean break.

Bedding replacement removes the bedding microbiome, organic reach environment and resets the periodic cycle.

Rule 6. High frequency of clean breaks can lead to extinction exclusively environmental microbes.

More frequent cage bedding replacements will prevent enrichment of microbes within the bedding that may alter the gut microbiome of the housed animal if bedding is ingested.

8.2 Physiological factors on organic and moisture content in bedding.

Although there are great body weight differences across mouse lines, [SPRET/Ei and CAST/Ei belonging to low body weight strains, SM/J strain to mid body weight group, AKR/J and KK/HlJ belonging to high body weight strains, and Swiss Webster very large body weight strain], in average the weight also depends on age, obesity, disease status and pregnancy. In average a mouse weighs 23.5 ± 0.9 g, n = 28. Adjusted to 30 g of body weight, **food intake** per day can range from 4.3 to 8.5 g/day (average **5.7 ± 0.2 g/day**). **Water intake** ranges from 6 to 11.8 ml/mouse per day, with average of **7.7 ± 0.3 ml**/30 g body weight/day. The Average **urine output** per mouse is ~**1.6 ml per day**, and the average water intake is ~ 5 ml per 24 hour period. But male house mice excrete urine at a rate 1.5-2.0 times that of females. Of relevance Females in estrus produce more urine than females in diestrus. Urine output per day increases during the latter two thirds of pregnancy and remains high throughout lactation. Density does not influence urine output per day for either sex over the range of densities tested. Castration reduces urine output per day in male mice, but ovariectomy in females does not alter rates of urine production. Dominant males produce more urine than subordinate males, but there are no similar effects for female mice https://www.ncbi.nlm.nih.gov/pubmed/24233678. The feces and urine has a great protein content, for some concentrations can be 1 million times higher that the MHC proteins in the urine. https://www.ncbi.nlm.nih.gov/pubmed/11740558 *Fluid spillage* from drinking water is in excess of **0.1 ml per day**. http://www.informatics.jax.org/mgihome/other/mouse_facts1.shtml https://www.ncbi.nlm.nih.gov/pmc/articles/PMC1397713/

8. R Code for Visualization of Cyclical Dynamics

Manuscript Figure 4g was generated using simulated data derived from the following functions ran in R studio Version 1.0.143 – © 2009-2016 RStudio, Inc. Mozilla/5.0 (Macintosh; Intel Mac OS X 10_12_6) AppleWebKit/603.3.8 (KHTML, like Gecko). The R version used was 3.4.0 (2017-04-21) – “You Stupid Darkness” Copyright (C) 2017 The R Foundation for Statistical Computing Platform: x86_64-apple-darwin15.6.0 (64-bit). The deSolve version was 1.20.

```
r <- 0.5; K <- 100; yini <- 0; p <- 40; M <- 1 #M is mice n per cage. p fecal pellets/day
qpellets <- function(t, y, parms)
list((p *M))
library(deSolve)
times <- seq(from = 0, to = 10, by = 1)
out <- ode(y = yini, times = times, func = qpellets, parms = NULL)
head(out, n=3)
M <- 2
out2 <- ode(y = yini, times = times, func = qpellets, parms = NULL)
M <- 3
out3 <- ode(y = yini, times = times, func = qpellets, parms = NULL)
M <- 4
out4 <- ode(y = yini, times = times, func = qpellets, parms = NULL)
M <- 5
out5 <- ode(y = yini, times = times, func = qpellets, parms = NULL)
plot(out, out2, out3, out4, out5, lwd = 2, main = "Accumulaton of feces vs mice")


bedbatch <- function(time, y, parms){
with(as.list(c(y, parms)), {
f <- r * S / (ks + S)
dS <- - 1/Y * f * N
```

```
dN <-        f * N
return(list(c(dS, dN)))
})
}
y <- c(S = 10, N = 5e4)
parms <- c(r=1.5, ks=5, Y=1e6, S0=10)
times <- seq(0, 40, 0.1)
####out <- ode(y, times, batch, parms)
####plot(out)
etime <- seq(10, 40, 10)  # time points to triger events
D = 0.95
eventfun <- function(t, y, parms) {           # event function
with(as.list(c(y, parms)), {
return(c(D * S0 + (1-D) * S, (1-D) * N))   # D = dilution rate; 0.1
})
}
out <- ode(y, times, bedbatch, parms,
       events = list(func = eventfun, time = etime))
nf <- layout(matrix(c(1,1,0,2), 2, 2, byrow = TRUE), respect = TRUE)
plot(out, col= "red")
```

9. Conclusion

The growth of bacteria in liquid media has been long modelled using various approaches, of which logistic growth curves have been the best studied. Here we used recently developed R package deSolve capabilities to easily incorporate dilution events to the logistic function to visualize cyclical events and the cummulative effects over time. Our bedding derived data presented in **Manuscript Supplementary Figure 10a-b** supports the use of the validated functions here described. Data indicate that succesful aerobic microbes would have primarily steady growth curves (the left half of an S-shaped logistic growth curve) over the life of a bedding cycle (period between intervals mice are switched from a dirty soiled bedding to the next new cage/bedding) as nutrients are constantly added to the bedding due to mice daily activities and biological functions (grinding of diet and defecation/urination/drinking habits). The deSolve R scripts here described assisted us to visualize numerous possibilities before conducting validating experiments depicted in **Manuscript Figures 5** and **6**. Ongoing efforts will integrate the mathematical nature of cyclical nutritious enrichment of bedding material into the model.

10.References

1. Karline, S., Petzoldt, T, Setzer RW. *Solving Differential Equations in R: Package deSolve.* Journal of Statistical Software. February 2010, Volume 33, Issue 9.

2. Karline, S., Petzoldt, T, Setzer RW. deSolve documentation at http://desolve.r-forge.r-project.org.

3. Drickamer LC. *Rates of urine excretion by house mouse (Mus domesticus): Differences by age, sex, social status, and reproductive condition.* J Chem Ecol. 1995 Oct;21(10), 1481-93.

4. Hurst JL1, Payne CE, Nevison CM, Marie AD, Humphries RE, Robertson DH, Cavaggioni A, Beynon RJ. *Individual recognition in mice mediated by major urinary proteins.* Nature. 2001 Dec 6;414(6864), 631-4.

5. Bachmanov A, Reed DR, Beauchamp GK, Tordoff MG. *Food Intake, Water Intake, and Drinking Spout Side Preference of 28 Mouse Strains.* Behav Genet. 2002 Nov; 32(6), 435–443.
